# Bacterial Siderophore Production in Metal-Rich Environments: Underexplored Sources of Siderophores and Insights into Bioremediation

**DOI:** 10.1021/acs.jnatprod.5c01586

**Published:** 2026-03-02

**Authors:** Madison Knapp, Lesley-Ann Giddings

**Affiliations:** Department of Chemistry, 6089 Smith College, Northampton, Massachusetts 01060, United States

## Abstract

Siderophores are iron-chelating secondary metabolites that increase the bioavailability of the essential nutrient iron. These molecules have several diverse applications in agriculture, pharmaceuticals, and bioremediation. Their diverse and inherent properties have made them ideal targets for natural product characterization using culture-dependent and culture-independent methods that often combine cell cultivation with genomic analyses. However, despite decades of work characterizing these molecules, there is a dearth of information concerning siderophore biosynthesis in metal-rich environments, such as acid mine drainage sites, volcanic ash, and other sources of metal pollution that are not deficient in iron. This Review focuses on bacterial siderophore biosynthesis, regulation, and transport as well as the roles of these metabolites within metal-rich environments. The effects of noniron metals on siderophore production are discussed in addition to the methods and challenges used to investigate and leverage siderophore biosynthesis for sustainable environmental and agricultural practices. The examples discussed underscore the need for metal-rich environments to be further explored for the identification of novel siderophores and siderophore-producing organisms that can be exploited for human use.

## Graphical abstract

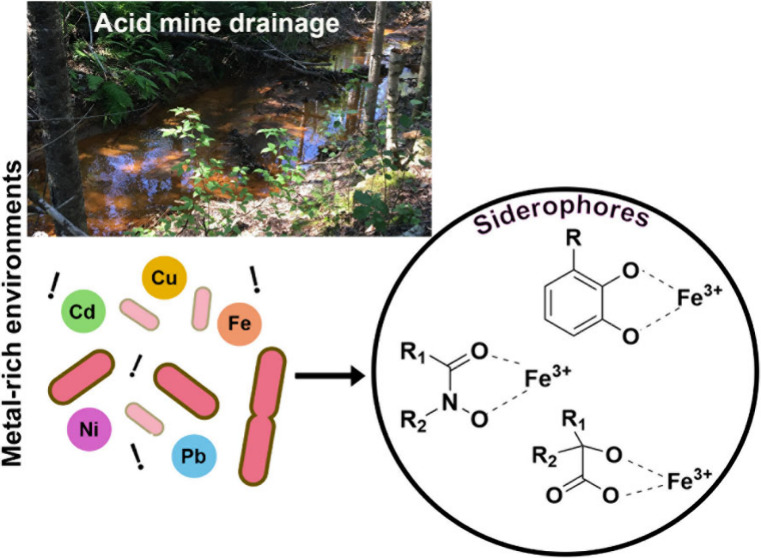

## Introduction

The term “siderophore” is Greek for iron (sidero-) carrier (-phore).^1^ These low-molecular-weight molecules are produced by plants, microbes, and some marine organisms to sequester iron, an essential nutrient for cellular metabolism.^2^ Iron serves as a cofactor for many enzymes and is involved in major biological processes, including the citric acid cycle, electron transport chain, oxidative phosphorylation, reactive oxygen species (ROS) detoxification, nitrogen fixation, biofilm formation, and aromatic amino acid biosynthesis.^3^ While iron is one of the most abundant metals within the Earth’s crust, its bioavailability can be limited.^4^ The physiologically relevant forms of iron are ferrous iron and ferric iron, the more biochemically utilized form. Ferric iron is significantly less soluble (∼10^–18^ M) at neutral pH than ferrous iron due to the formation of insoluble ferric hydroxides.^5,6^ Most bacteria require a ferric iron concentration of at least 10^–6^ M; thus, siderophores improve the bioavailability of ferric ions by selectively and tightly binding to these ions with very low *K*_d_ values (e.g., 10^–52^), allowing these organisms to regulate ferric iron concentrations.^7,8^ Iron levels must be strictly regulated, as a surplus can generate ROS and increased Fenton chemistry; likewise, a deficiency can result in metabolic dysfunction.^4^ Once a siderophore is bound to ferric iron, the complex crosses cell membrane via permeases or transporter proteins, ferric iron is then reduced to ferrous iron, and the metal is released from the complex.^9,10^

Beyond delivering iron to the cell, siderophores attract attention in research because many are bioactive secondary metabolites widely used in medicine and agriculture.^11,12^ Several siderophores exhibit antimicrobial activity and are used in metal chelation therapies in the clinic.^13^ For example, desferrioxamine B, sold as Desferal, is a clinically approved drug produced via microbial fermentation for the treatment of iron overload disorders and metal poisoning, including aluminum.^14,15^ Cefiderocol, sold as Fetroja, is also a clinically approved antibiotic used for the intravenous treatment of Gram-negative bacterial infections. This siderophore-containing antibiotic functions as a Trojan horse by using the iron-siderophore transport system for bacterial uptake.^13^ In addition to their biological functions, siderophores can also exhibit antimalarial, photolytic, anticancer, immunosuppressive, and neuroprotective activities and are utilized in medical imaging.^15–20^ In agriculture and environmental science, siderophores are used to recover, sequester, or filter metal from soil and other materials.^21^ The broad application of siderophores across many fields makes them desirable targets for studies probing their biosynthesis, regulation, and uptake.^13^

Most reviews on siderophores highlight molecules produced in iron-deficient environments, such as during pathogen infections in human hosts, where free ferric iron is limited, activating the transcription of siderophore biosynthetic genes.^13,22^ However, this Review aims to highlight the production and function of bacterial siderophores in iron-rich and other heavy metal-contaminated environments. Bacterial siderophores are more commonly reported than other microbial siderophores, especially in these extreme environments.^1,23^ Furthermore, increases in the concentrations of other heavy metals, such as zinc, copper, and rare earth elements, even in the presence of iron, have increased siderophore production in some instances.^24^ These metals bind to siderophores with various affinities, impacting the environmental roles of the siderophore-metal complexes, which can be utilized to clean up metal pollutants.^24^ Metal-rich environments are underexplored and increasing in number due to anthropogenic activities, such as mining, metallurgy, agricultural practices, waste disposal, energy production, transportation, and the production of microelectronics.^25,26^ Thus, understanding bacterial siderophore production in metal-rich environments can provide access to novel molecules and insight into how to bioremediate these toxic locales.^27^

## Siderophore Classes and Biosynthesis

Ferric iron is coordinated by a limited number of chemical ligands, such as carboxylates, hydroxamates, phenolates, catecholates, and heterocyclic oxazoline and thiazoline groups, typically forming six-coordinate complexes.^28^ Thus, the following four main classes of siderophores are defined by their metal-binding ligands: hydroxamates (e.g., desferrioxamine B/E), thiazoline/oxazolines (e.g., pyochelin), hydroxycarboxylates (e.g., vibrioferrin), and catecholates (e.g., enterobactin) (Figure 1).^28^ These water-soluble molecules facilitate iron solubility and uptake from the environment by chelating proximal heteroatoms.^29^ Each class of siderophores has distinct molecular properties that dictate metal ion selectivity.^29^ There are also mixed classes, as the structural frameworks used to synthesize these molecules can be combined (e.g., pyoverdine) (Figure 1).^30,31^

**Figure 1:**
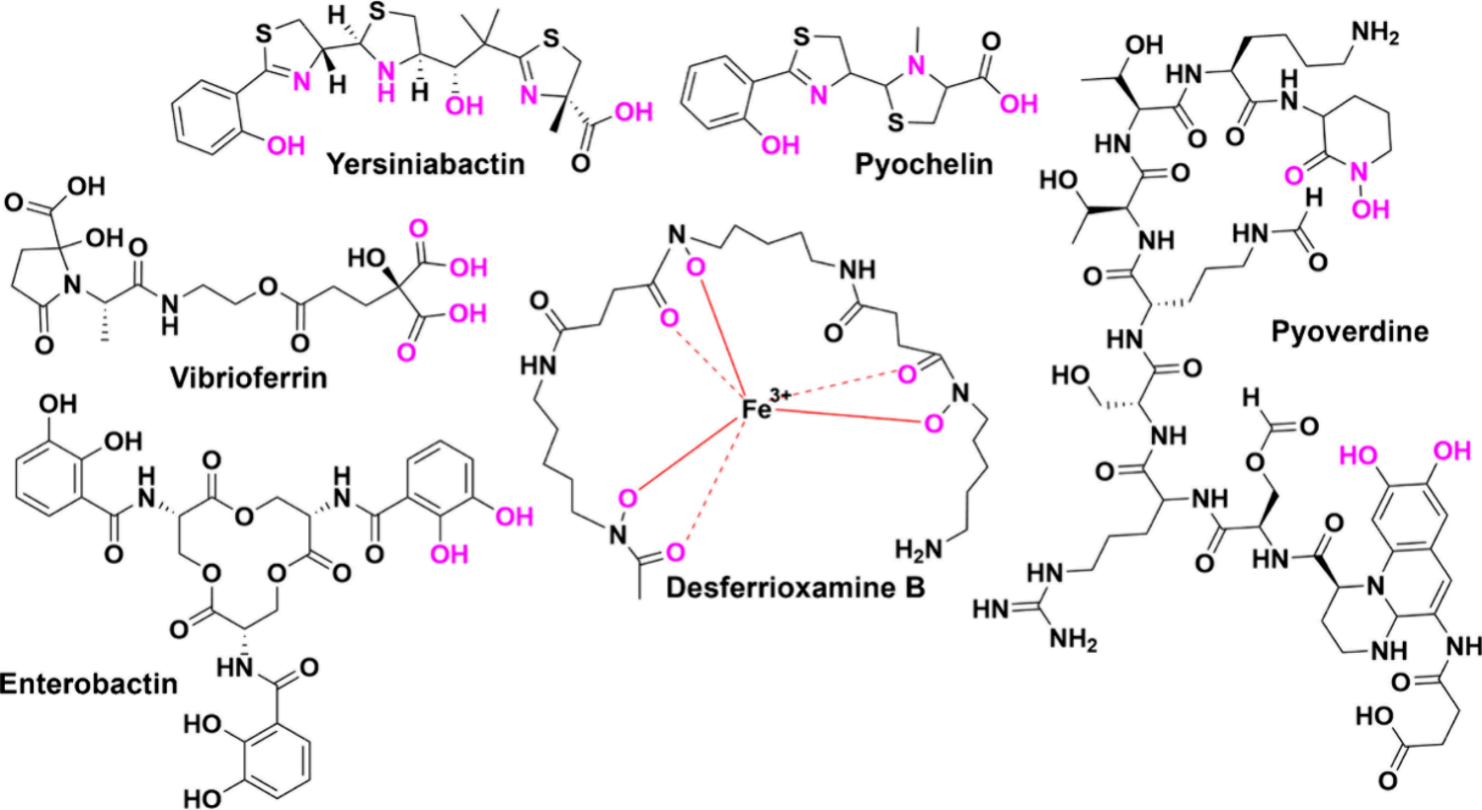
Various siderophores that chelate ferric iron. The functional groups highlighted in pink bind to ferric iron.

Siderophores are synthesized by either nonribosomal peptide synthetase (NRPS)-dependent or NRPS-independent synthase (NIS) biosynthetic pathways, both of which require the adenylation of substrates to activate the amino acid for peptide bond formation.^32^ NRPS-dependent siderophores are composed of amino acids linked via peptide bonds, which are formed by a multicomponent enzyme (NRPS) involved in the synthesis of many natural products.^17^ There are three categories of NRPS enzymes: linear (type A), iterative (type B), and noniterative (type C).^33^ Siderophores have been reported in bacteria and fungi from all three categories.^34–36^ The linear NRPS (type A) consists of three nonreusable modules joined in a single polypeptide chain that catalyze the initiation, extension, and termination reactions (Figure 2A).^37^ The initiation module consists of an adenylation (A) domain and a peptidyl carrier protein (PCP) domain, which contains a 20 Å long phosphopantetheine prosthetic group ending with a thiolate to tether substrate.^33,38^ Adenylation facilitates peptide bond formation once the thiolate of the phosphopantetheinyl prosthetic group in the PCP domain attacks the electrophilic carbonyl on the A domain.^37^ The extension module consists of at least the A, PCP, and condensation (C) domains.^37^ After the carboxylate of a new amino acid is activated via adenylation and loaded onto the PCP domain, the C domain catalyzes N-to-C terminal condensation between the PCP-acceptor substrate and the PCP-growing peptide in the extension module.^37^ Additional tailoring domains that perform methylation, oxidation, cyclization, or epimerization can also be found in this module.^37^ The termination module consists of domains found in the extension module, followed by a thioesterase (TE) domain at the C-terminus.^33^ The TE domain releases the growing peptide from the final PCP domain by forming an oxoester bond via an active site serine attacking the thioester linkage of the peptide attached to PCP.^37^ Finally, the TE domain hydrolyzes the oxoester, releasing the peptide from the NRPS.^17^ Postassembly line modifications, such as glycosylation, lipidation, or halogenation, can occur after the release.^39^ The iterative (type B) NRPS reuses the same domains multiple times, and the nonlinear NRPS (Type C) does not synthesize siderophores in a manner that correlates to the arrangement of modules.^40^ Yersiniabactin, pyochelin, and enterobactin are examples of siderophores produced via NRPS-dependent pathways (Figure 1 and 2A).^41–43^ These biosynthetic pathways can also involve a polyketide synthase, another multicomponent enzyme, that links acyl groups to an internal cysteine residue via a condensation reaction.^33,44^

**Figure 2:**
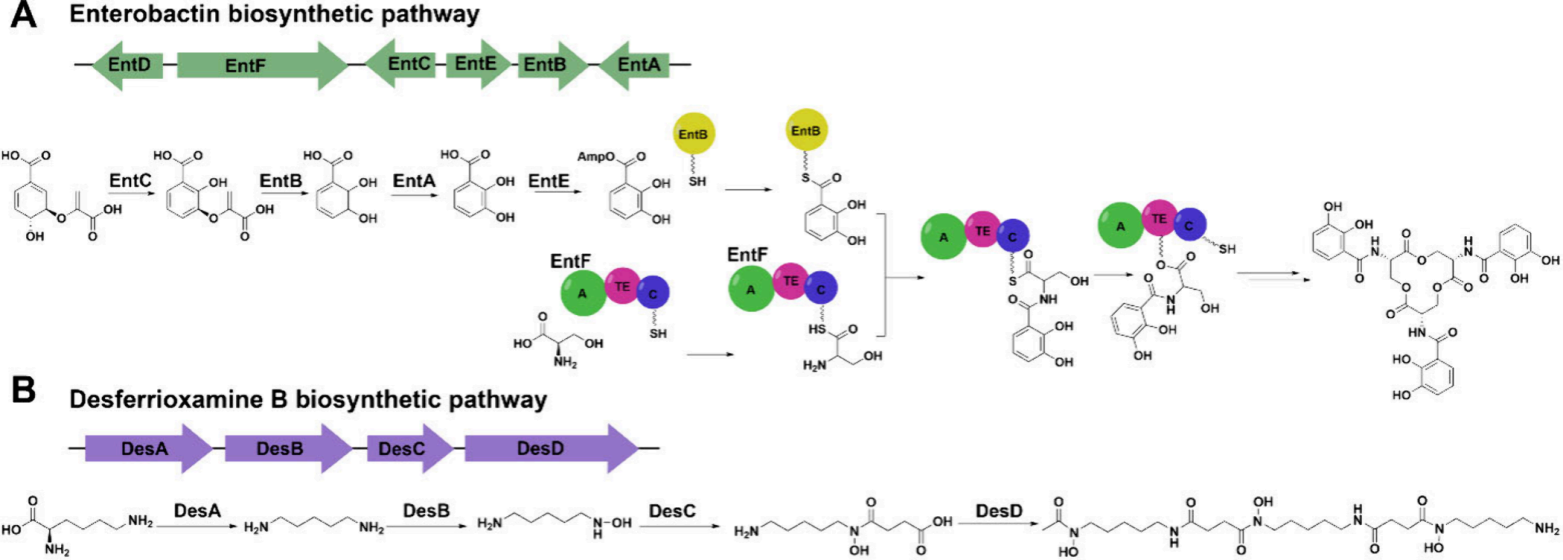
Examples of NRPS and NIS siderophore biosynthetic pathways. (A) Enterobactin is biosynthesized from the NRPS-dependent pathway, requiring the multicomponent enzyme for multiple transformations of the substrate. (B) Desferrioxamine B is produced by the NIS pathway, where each step is catalyzed by a distinct enzyme.

Unlike the NRPS-dependent pathway, enzymes in the NIS pathway are not well characterized; thus, their function is mostly hypothesized.^28^ The NIS pathway catalyzes a single enzymatic reaction between carboxylates (e.g., citric acid) and amines (e.g., lysine) to form the final siderophore product using ATP as a cofactor to adenylate carboxylate groups in substrates (Figure 2B).^45^ Prior to this step, modifications can be made to the amine, such as decarboxylation and *N*-hydroxylation, via other pyridoxal phosphate-dependent decarboxylases and flavin-dependent *N*-hydroxylase enzymes.^46,47^ Three major superfamilies of synthases catalyze condensation reactions between specific substrates, including type A (citric acid and monoamine or amide substrates), type B (α-ketogluturate and lemonamine), and type C (succinyl or citric intermediates with monoamine or amide substrates).^48^ Aerobactin is synthesized using type A and C NIS enzymes, and the desferrioxamines are synthesized via type C enzymes (Figure 2B).^45^ NIS enzymes can also function in conjunction with other enzymes in NRPS-dependent pathways to produce siderophores.^45,49^ For example, petrobactin is synthesized from a hybrid of NIS- and NRPS-dependent pathways to produce two catecholates and a hydroxycarboxylate ligand that bind iron.^50^ The genes involved in the NIS pathway can be challenging to identify because of the lack of experimentally validated homologous comparisons in model organisms, resulting in misannotation and the “overprediction” of molecular function.^51^ However, these issues can be resolved using bioinformatic tools to predict and identify new NIS biosynthetic gene clusters, as well as collecting experimental genomic reconstruction data.^52–55^

### Bacterial Siderophore Regulation and Iron Transport

The regulation of siderophore biosynthesis under iron-deficient conditions is tightly controlled at the transcriptional level to maintain iron homeostasis and pathogenicity, as well as conserve energy.^56^ These pathways are regulated by genes involved in iron sensing, chelation, transport, and release, which are generally under the control of a universal iron-dependent repressor protein.^57^ The regulation of siderophore biosynthesis in bacteria typically relies on either the ferric uptake repressor (Fur) or diphtheria toxin regulator (DtxR).^58–60^ Despite differences in sequence, in the presence of sufficient iron levels, both regulators first bind ferrous iron to an iron binding site, which undergoes a conformational change to allow the repressor protein to bind to specific DNA sequences (e.g., 19-bp sequence Fur boxes) involved in iron acquisition, preventing their transcription.^61^ Under iron-limiting conditions, ferrous iron is released, and transcription regulatory enzymes undergo a different conformational change that prevents the transcriptional promoters of genes involved in iron acquisition from being blocked, increasing the biosynthesis of iron uptake genes.^57^

Although Fur and DtxR are the primary iron regulators for many bacteria, additional layers of control exist, making the regulation of siderophores a complex process.^62^ Siderophore biosynthesis can be activated by two-component signal transduction systems, sigma factors, and Ara-C type and other transcriptional regulators, many of which are regulated by Fur.^57^ For example, several two-component signal transduction systems have been implicated in the regulation of the synthesis and transport of siderophores, such as EnvZ/OmpR in *Escherichia coli*, as well as AlgZ/AlgR, GacS/GacA, PfeR/PfeS, and HK/RR in *Pseudomonas*.^63–67^ Aside from one and two-component systems, the extracytoplasmic function (ECF) sigma factor family is the third most abundant bacterial signal transduction system involving a three-component signaling cascade.^57,68,69^ In *E*. *coli,* ferric dicitrate binding to the outer membrane receptor FecA triggers a signaling cascade through the transmembrane protein FecR, releasing the ECF sigma factor FecI, and activating the transcription of ferric-citrate (*fec*) transport genes.^70^ Beyond sigma factors, bacteria also use AraC-type transcriptional regulators, which are fusion proteins of AraC DNA-binding and iron-siderophore substrate-binding domains that sense siderophores before or after iron uptake.^57^ For example, (des)­ferrioxamine was reported to stabilize the binding of the *foxA* regulatory region to FoxR, an AraC-type regulator of ferrioxamine uptake in *Salmonella.* ^71^ Other post-transcriptional and post-translational mechanisms mediated by small regulatory RNAs (e.g., RyhB in *E. coli*) and riboswitches can regulate siderophore biosynthesis to ensure iron homeostasis.^72–74^ Virulence genes and other signals can also modulate the production of siderophores for organisms to adapt to their changing environment.^57^ Thus, the regulation of siderophores is a multifaceted and complex process, as membranes and receptor proteins play integral roles in regulation as iron crosses these barriers to enter cells.^75^

Once siderophores are synthesized, these iron-free molecules are secreted into the environment alone or bound to proteins to aid in iron capture and transit (Figure 3).^1,76,77^ In some instances, siderophores can diffuse away and be highjacked by surrounding siderophore non-producing organisms, compromising the fitness of the siderophore-producer.^1,5^ However, mechanisms for transporting siderophores bound to proteins are more well understood.^78^ Genes encoding transport proteins or systems are typically clustered with siderophore biosynthetic genes within bacterial genomes.^79^ Transport (e.g., efflux and uptake) proteins belong to the major facilitator superfamily (MFS), the resistance, nodulation, and cell division (RND) superfamily, and the ATP-binding cassette (ABC) superfamily.^57^ These superfamilies use secondary transporters and multicomponent systems driven by ion gradients, as well as primary active transporters that use ATP hydrolysis.^80^ These transport proteins differ between Gram-positive and Gram-negative bacteria because Gram-positive bacteria lack an outer membrane to translocate siderophores.^81^

**Figure 3:**
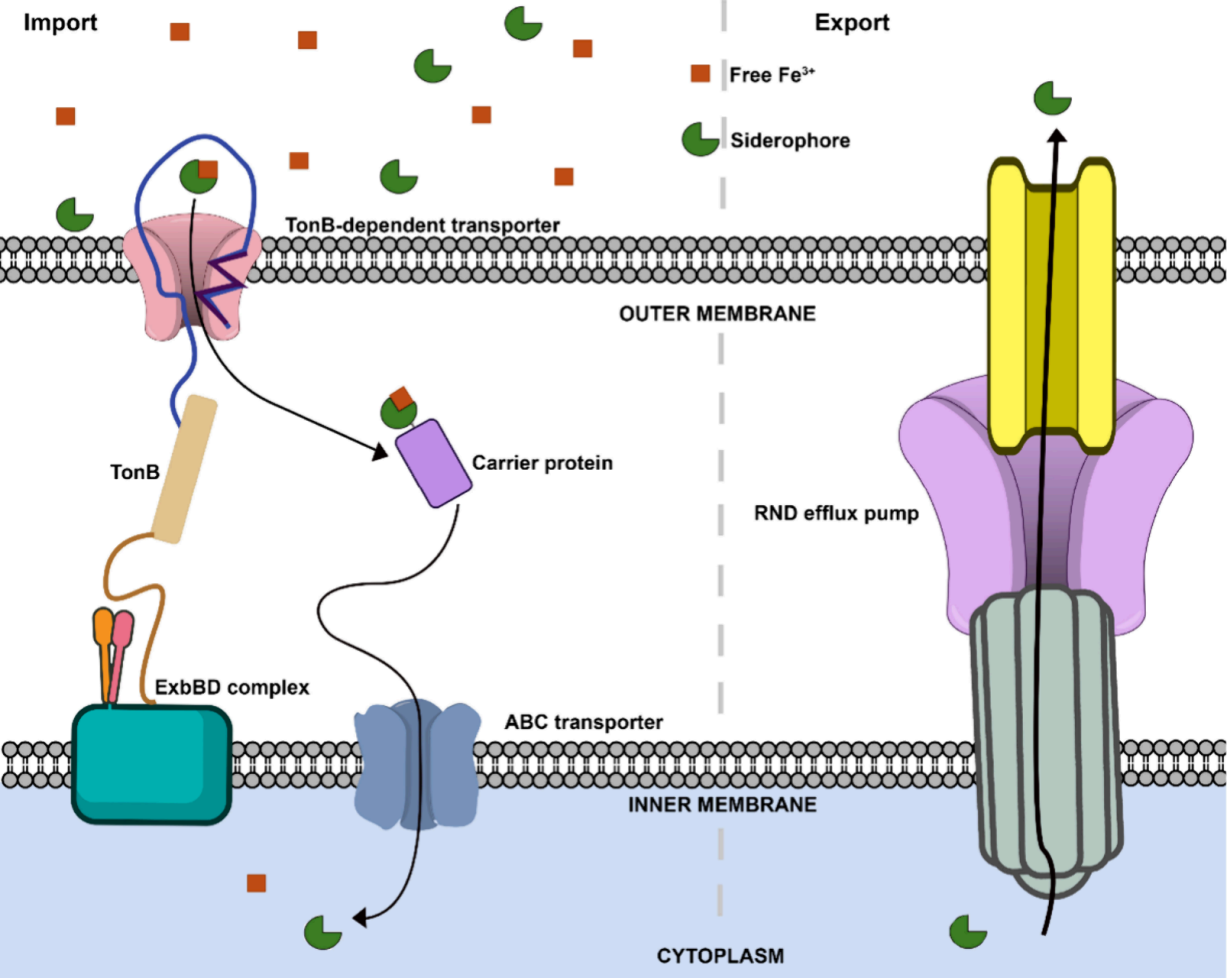
Siderophore import (left) and export (right) pathways in Gram-negative bacteria. The following three steps are involved in import: (1) transport of iron-bound siderophore through the outer membrane into the periplasm; (2) the TonB-dependent transporter transfers the complex to the carrier protein; and (3) interactions between the carrier protein and ABC transporter to cross the inner membrane for final reduction and release of iron ions into the cytoplasm. Siderophore export relies on the RND efflux pump to cross both membranes for the final release of the siderophore outside of the cell.

Siderophore transport in Gram-negative bacteria is better understood.^57^ Export involves transporter and energy-transducing proteins carrying siderophores across the outer and inner membranes.^82^ For example, in *P*. *putida*, both tripartite ABC transport systems and RND efflux systems that utilize transport proteins and protein channels move siderophores, such as pyoverdine, from the cytosol to outside the cell (Figure 3).^76,83,84^ However, these systems are not universal for all Gram-negative bacteria, as they can be species-dependent, and alternative efflux systems, including MFS efflux systems, can be used under select conditions.^84–87^ Import into the periplasm typically involves the TonB-dependent receptors interacting with the TonB system, which consists of the following complex of inner-transmembrane proteins: TonB (coupling protein), as well as ExbB and ExbD (inner membrane energy transducers).^88,89^ Once in the periplasm, the siderophore-iron complex dissociates from the TonB-dependent receptor and binds to a carrier protein to be delivered via ABC transporters or additional permeases into the cytosolic membrane (Figure 3).^84,89,90^ The ABC transporters that span the inner membrane hydrolyze ATP upon binding to the periplasmic carrier protein bound to the siderophore-iron complex.^91^ This interaction triggers the reduction of ferric iron to ferrous iron, which is now available for metabolic processes within the cell.^91–93^

Siderophore transport in Gram-positive bacteria mainly involves ABC transporters and MFS-type efflux pumps, as RND cell efflux systems are characteristic of Gram-negative bacteria.^94,95^ There can be multiple transporters that recognize different types of siderophores.^96^ For example, ABC-type exporters, such as ExiT or IroC, have been shown to transport exochelin or enterobactin in *Mycobacterium smegmatis* and *S. enterica*, respectively.^97,98^ Secretion can also involve MFS-type efflux pumps, such as YmfE in *Bacillus subtilis*, used to secrete bacillibactin.^99^ Import in Gram-positive bacteria, such as *B. subtilis*, is simpler than that in Gram-negative bacteria due to the absence of an outer membrane.^100^ In Gram-positive bacteria, periplasmic siderophore-binding proteins anchored to membranes bind and deliver siderophore complexes to ABC transporters for the eventual release of iron into the cytosol.^5,101^ Although many Gram-positive bacteria, including the extensively studied *Staphylococcus aureus*, cause dangerous pathogen infections, much less is known about siderophore transport in these systems compared to that of Gram-negative bacteria.^101,102^

### Methods for Investigating Siderophores

Siderophores are commonly investigated using a combination of culture-dependent and culture-independent approaches.^103,104^ Culture-dependent methodology relies on the researcher’s ability to grow and cultivate organisms on the benchtop under conditions that mimic their native environments.^105,106^ Once an organism is cultivated, siderophore production can be detected using the Chrome Azurol S (CAS) assay, a colorimetric assay used to detect siderophores based on these metal chelators outcompeting CAS (blue dye) for binding iron, resulting in a color change from blue to yellow or orange.^103,107^ This liquid assay can be modified (e.g., the iron can be replaced by other metal ions) and applied as an agar overlay for culture plates.^108^ The CAS assay is widely used to visually detect siderophore production within hours without needing to know about the microbe or type of siderophore, other than a suitable growth condition.^109^ The siderophore type can be determined without isolation using colorimetric assays, such as Csaky, Arnow’s, and Shenker’s assays to identify hydroxamate, catecholate, and hydroxycarboxylate siderophores, respectively.^110–112^ Given that these assays do not require chemical isolation, several reports do not elucidate the structure of siderophores or only report known compounds with commercially available standards.^113,114^ Analytical methods, such as mass spectrometry (MS) or nuclear magnetic resonance, can be used to isolate and characterize siderophores when yields are sufficient.^51,52,115,116^ To identify siderophores, SIDERITE is a free database that contains over 800 known siderophore structures in SMILES format.^117^ This database can be used in conjunction with mass spectrometry analyses of environmental samples or microbial cultures to identify known siderophores.^118,119^ Metal-binding compounds can also be identified using other workflows that take advantage of public data sets, including the Global Natural Products Social molecular networking platforms.^120^

Culture-independent identification of siderophores harnesses the power of genomics and bioinformatic approaches.^104^ Genomic sequence information allows for the identification of biosynthetic gene clusters that encode genes responsible for the biosynthesis, transport, and regulation of siderophores, enabling the functional prediction of molecular structures.^104^ Advances in next-generation sequencing have made DNA sequencing not only faster with increased capacity but also more affordable and widely accessible.^121^ The high-throughput sequencing of RNA transcripts or transcriptomics is another method for identifying expressed siderophore biosynthetic genes and has been used to develop antivirulence targets.^122^ Furthermore, bioinformatic tools, such as antiSMASH, NRPSFinder, and PRISM, can be used to find and visualize gene clusters based on sequenced reference genomes.^55,104^ Additional downstream processing can also be performed using the Enzyme Function Initiative’s Enzyme Similarity Tool (EFI-EST), which groups proteins together in networks based on sequence similarity.^123^ Often, laboratories will use a combination of methodologies to identify and characterize natural products, but no one path is without its own limitations.^105^ Combinatorial approaches, including multiomics (genomics, transcriptomics, and metabolomics), are increasing in popularity as they provide natural product research a wealth of knowledge from high-throughput screens that can translate to success on the benchtop.^105^

### Ecological Roles of Siderophores

Using the aforementioned tools, siderophores have been reported to not only sequester ferric iron but facilitate complex interactions between organisms for iron acquisition within the environment.^1,24^ Within competitive environments, such as the soil surrounding plant roots or rhizosphere, siderophores are required to exchange or acquire essential nutrients.^124^ The secretion of siderophores can be detrimental to some cells, while being critical for others.^124^ The exchange of siderophores between producers and receivers is known as cooperative behavior, which depends on local receiving partners having the same receptors and transporters to uptake siderophore complexes.^1^ This behavior is well documented for *P. aeruginosa*, as it shares pyoverdine (Figure 1) with local non-producing organisms to increase the growth and cell size of receivers.^1^ Anammox bacteria found in wastewater are examples of nonsiderophore-producing receivers that perform anaerobic ammonium oxidation and rely on bacterial communities for ferric iron.^125,126^ This exchange is facilitated through interactions between anammox symbiotic bacteria that produce and deliver siderophores to anammox host bacteria.^127^ Of course, not all microbial interactions are positive, and in environments where two or more siderophore producers are growing, organisms can outcompete each other for iron, resulting in inhibited growth, iron starvation, and ultimately cell death.^128^ Thus, siderophores have multifaceted roles in the environment, where they are used as iron transporters, as well as mediators of interactions, especially between host and symbiote.^128^

Siderophores have mostly been explored in organisms inhabiting iron-deficient environments, such as sites of microbial infection, marine environments, or the competitive rhizosphere.^100^ The molecules are well-studied in human microbial infections, as free iron is scarce in the human body but required for pathogenic microbes to grow.^16,129,130^ Marine environments also rely on siderophore-producing bacteria to harvest metal ions from iron-deficient seawater. Iron levels are considerably lower (e.g., in the pM range for polar marine environments), than in iron-deficient conditions on land (>10 nM in soil).^131,132^ Siderophore producers have been widely explored in agriculture to promote plant growth under stressful conditions, such as overcultivated nutrient-deficient agricultural fields.^133^ Plants commonly use microbial siderophores to sequester iron from the environment by binding ferric iron secreted into the rhizosphere, reducing it into the more soluble ferrous form, and delivering ferrous iron back to plants via the root system.^134^

While commonly reported from iron-limiting environments, siderophore-producing organisms inhabit locales with a wide range of metal concentrations, including naturally occurring and anthropogenic heavy metal-polluted environments.^135^ Heavy metals are defined as a group of metals and metalloids in the Earth’s crust with high atomic numbers and densities greater than 4 × 10^6^ mg/L, such as cadmium, nickel, copper, cobalt, lead, arsenic, zinc, and mercury.^136^ The ongoing increase in heavy metals within our biosphere is detrimental because these elements are not biodegradable and toxic to lifeforms.^137^ At high concentrations, heavy metals can form ROS and free radicals that target DNA, lipid, and protein structures, resulting in cell damage, loss of cellular functions, or even cell death.^137,138^ Despite the inherent toxicity of these environments, such as mining sites that generate acidic and metal-rich effluent, there are microbes that persist under these conditions, with some requiring heavy metals as nutrients and for metabolism.^139,140^ Siderophores produced by these microbes, bacteria being the most commonly reported, play major roles in heavy metal-rich environments by reducing metal toxicity while facilitating metal transport.^135^ Thus, investigating siderophore production in these underexplored environments is critical for advancing our understanding of their environmental roles and better informing their application.^135^

### Metal Chelators in Metal-Rich Environments

To date, acid mine drainage sites are among the most documented metal-contaminated environments (Figure 4A).^141–143^ Acid mine drainage is an acidic, metal-rich discharge produced from the oxidation of metal sulfides in mining regions and further accelerated by microbial metal oxidation activities.^144^ These sites can be rich in iron and other metals and yet siderophores are still produced; hence, their production is not limited to metal-deficient conditions (Figure 4B).^24,145^ Figure 4A shows the distribution of active mining sites and rare earth element deposits in the United States, underscoring the abundance of these sites.^141,142^ CAS and other colorimetric assays have been routinely used to detect siderophore-producing bacteria in several mining sites. For example, CAS detected an increase in siderophore-producing taxa, such as *Pseudomonas*, along a natural heavy metal gradient in a poly metallic mining area in Cornwall, United Kingdom.^24^ In addition to iron, the siderophores released into metal-rich environments can bind to various heavy metals, improving survival under harsh conditions. In a lead and zinc mine in Shangyu, Zhejiang, China, a strain of *Burkholderia* was reported to produce a catecholate-like siderophore that solubilized ferric iron as well as zinc­(II), copper­(II), and cadmium­(II).^113^ Siderophores from multiple strains of *Pseudomonas* isolated from an acid mine drainage site in Slippery Rock, Pennsylvania, USA, chelated iron in addition to several rare earth elements, such as praseodymium, scandium, and europium.^146^ In this study, researchers also showed that acid mine drainage sites had a higher content of siderophore-producing bacteria than in other samples collected at a noncontaminated site.^146^ Although most siderophores from mining environments have been found in acid mine drainage sites, siderophore biosynthetic genes have also been reported in alkaline mining environments, such as phosphate mine wastes at Kettara Mine in Morocco.^147^ Thus, siderophores can be produced in any metal mining environment.

**Figure 4:**
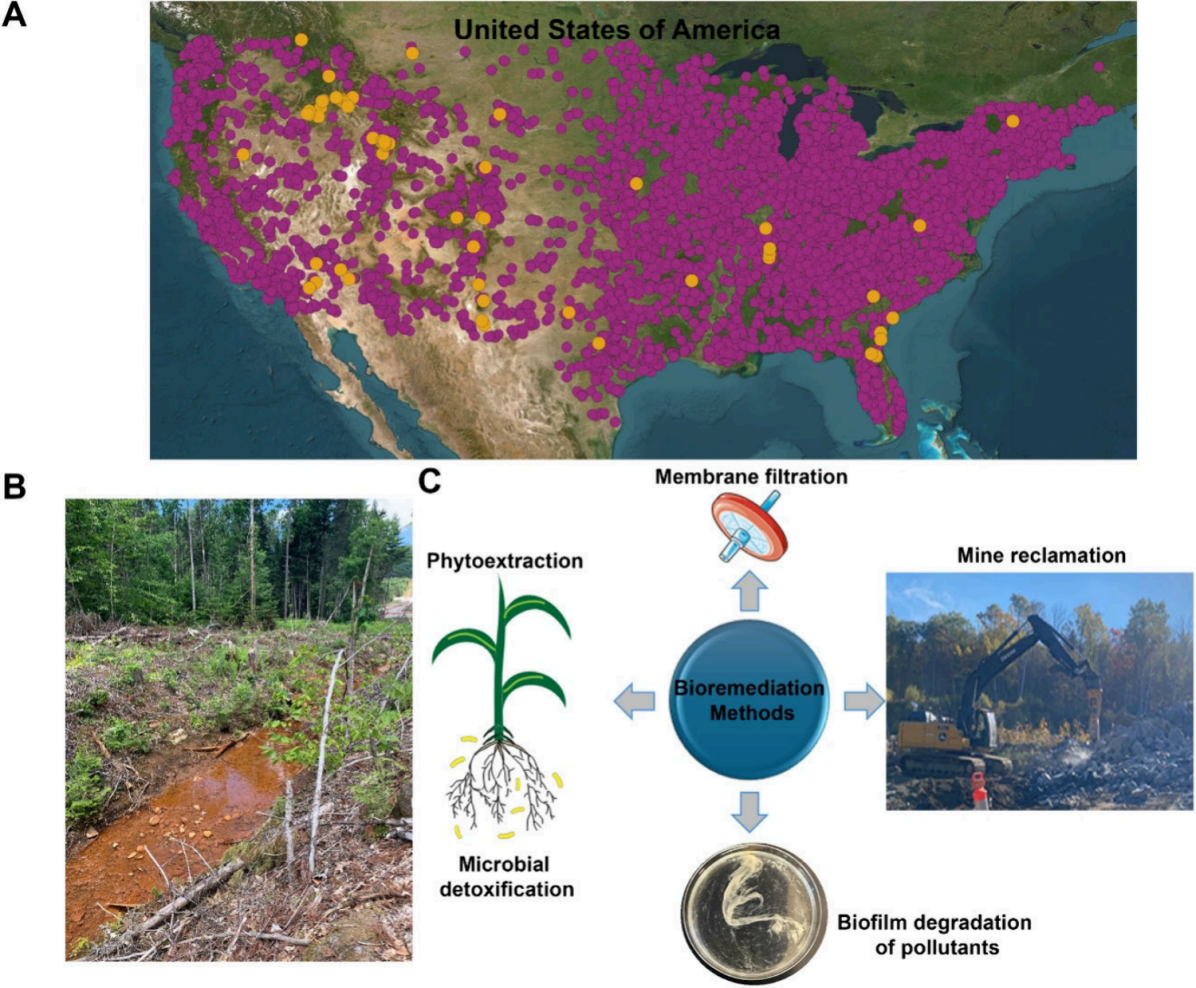
Metal-contaminated sites are abundant and often the targets of bioremediation efforts due to the toxicity of high concentrations of metals to living organisms. (A) A map of the continental United States, where the purple circles represent active mines and the orange circles represent rare earth element deposits. Coordinate information was retrieved from https://mrdata.usgs.gov/ree/ and https://mrdata.usgs.gov/mineplant/ and visualized using QGIS software. (B) Water runoff from Ely Copper Mine, an abandoned copper mine in Vermont, USA, is an example of an acid-mine drainage site that is currently undergoing abiotic remediation. (C) Common methods for bioremediation.

The roles of siderophores in metal-contaminated environments involve metal mobilization, solubilization, and precipitation in addition to increasing the metal tolerance of organisms.^148^ While still having a preference for iron, desferrioxamine B (Figure 1) can dissolve and transport rare earth elements in volcanic ash particles to river waters under siderophore-rich conditions due to limited dissolved iron.^149,150^ Bau et al. suggest that siderophores mobilize rare earth elements, specifically cerium, during weathering events that form soil on the Earth’s surface.^149^ Pyoverdine and pyochelin (Figure 1) have been shown to sequester aluminum­(III), cobalt­(II), copper­(II), nickel­(II), lead­(II), and zinc­(II) from extracellular media, with pyoverdine also binding europium­(III) and terbium­(III), suggesting that these siderophores contribute to the heavy metal resistance to *P. aeruginosa* PA01.^151^ Although various siderophores can bind additional metals, including lanthanides (e.g., cerium­(III), neodymium­(III), gadolinium­(III), and ytterbium­(III)), the complexes they form are typically less stable than those with ferric iron.^13,152^ However, the stability of these complexes can be improved under selective conditions.^153^ Under redox oxic conditions, desferrioxamine B complexes with cerium­(III) with a stability constant consistent with that observed with ferric iron.^153^ Other factors, such as pH, can also impact the stability of these complexes.^135^ Thus, environmental conditions affect the role of siderophores in metal-rich environments.^149^

Other natural product metal-chelating agents or metallophores have been found in mining environments using CAS-modified screens.^154^ For example, the taiwachelin-producer *Cupriavidus basilensis* BL-MT-10 was isolated from a heavy metal-contaminated environment in Montana, USA, using agar plates supplemented with copper­(II) or cerium­(III) ions, and metallophore production was confirmed by a CAS overlay assay.^154^ Methane-oxidizing bacteria have also been reported to produce the metallophores methanobactin and pseudopaline, which preferentially complex to copper and zinc, respectively.^155^ These metallophores play crucial environmental roles, such as helping microbes scavenge metal ions for detoxification.^156,157^ For example, *Delftia acidovorans* is a Gram-negative bacterium found in the biofilms of mined gold nuggets that produces delftibactin A, an NRPS-synthesized peptide containing hydroxamate moieties that bind gold­(III) to form inert gold nanoparticles for detoxification.^156^ Species of *Delftia* and *Pseudomonas* have been reported to produce metallophores in response to arsenic­(III), arsenic­(V), zinc­(II), and nickel­(II) in other heavy metal-contaminated sites, such as the polluted Lerma–Chapala Basin, likely impacting metal transport and reducing the toxicity of these compounds within the environment.^157^ Thus, metal chelation is prevalent in metal-rich environments and contribute to the overall survival of organisms in these extreme conditions.^135^

While metallophores, including siderophores, are not widely reported in most acid mine drainage studies, genome sequencing has facilitated the identification of a multitude of heavy metal resistance genes in addition to NRPS gene clusters.^158^ Heavy metal resistance genes typically cluster with siderophore-biosynthetic genes within bacterial genomes, which is very useful due to the challenges in predicting the structure of a metabolite product or gene function for a novel biosynthetic gene cluster based solely on gene content.^159^ For instance, the delftibactin A biosynthetic gene cluster was determined to be flanked by a tripartite heavy metal efflux pump, supporting the initial hypothesis of the role of delftibactin A in detoxification.^156^ NRPS genes can be identified based on conserved sequence motifs, but without functional data, predicting gene function can be challenging due to their dissimilarity to known biosynthetic genes or differences in the GC content of organisms.^158^ Several heavy metal resistance genes have been identified in heavy metal-contaminated microbiomes, indicating a great potential for these environments to produce siderophores.^158^ Genomic sequencing performed on acid mine drainage and isolated bacteria collected from the Superfund site, Ely Copper Mine, in Vermont, USA, revealed the presence of over 200 putative metal resistance genes, such as *copR*, *czcR*, and *wtpC*, which play a role in copper, zinc/cadmium/cobalt, and tungsten/molybdenum resistance, respectively (Figure 4B).^139,160^ Several NRPS and siderophore transporter genes were detected, but no dedicated siderophore biosynthetic gene clusters could be annotated.^160^ In fact, a large percentage of the Ely Copper Mine metagenome could not be annotated, as there was no homologous information for these microbial sequences in databases, leaving many open questions about the potential secondary metabolites that could be produced within this environment.^160^ As such, these sites are becoming increasingly mined for organisms that can be used to remediate metal pollution using microbial detoxification and other methods (Figure 4C).^161^

### Effects of Heavy Metals on Siderophore Production and Environmental Implications

Siderophores in metal-contaminated environments raise questions of how siderophore production is induced and whether certain metals can modulate siderophore biosynthesis with or without iron.^24,162,163^ Several studies have shown that the presence of certain metals increases siderophore production, although whether specific metals, organisms, or other environmental conditions induce this production remains unclear.^24,162,164,165^ For example, when *P. aeruginosa* strain PA01 was cultured in media supplemented with iron and spiked with either copper­(II) or nickel­(II), the production of both pyochelin and pyoverdine increased by over 200%.^24,151^ The same strain of *P. aeruginosa* was later found to produce more siderophores in heavy metal-contaminated soils than in uncontaminated environments.^24^ However, the authors suggested that this increase was due to the acidic nature of the soil selecting for siderophore-producing bacteria that can withstand a lower pH.^24^ Pyoverdine production has also been observed to increase in the presence of four lead-tolerant strains (50 mg/L of lead) of *P. aeruginosa* from lead-polluted water in Girardota, Antioquia, Colombia.^163^ In iron-depleted media, desferrioxamine E production increased when *Gordonia rubripertincta* CWB2 was cultured in the presence of aluminum, cobalt, gadolinium, germanium, neodymium, vanadium, or zinc, but some of these metals inhibited cell growth.^162^ The observed changes in siderophore production in metal-rich locales can be complex and unpredictable across organisms given their needs for survival.^24^ However, these studies underscore the need to understand how noniron metals impact siderophore biosynthesis under conditions that are not just nutrient-limiting.^148^

Once siderophores bind to noniron metals, the cellular processing of these complexes, initially evolved for iron, and the uptake of noniron metals is variable and depends on the siderophore, organism, and metal.^148^ Specific uptake pathways have been identified in *Yersinia pestis*, the cause of the bubonic plague in humans, which produces the siderophore yersiniabactin that binds ferric iron, zinc­(II), and copper­(II) (Figure 1).^166^ This organism has separate Yfe and ZnuABC uptake systems for the iron-bound and zinc-bound complexes, respectively.^166^ Zinc­(II) inhibits the ferric iron transporter Yfe by binding in place of ferric iron in the protein, whereas the zinc–siderophore complex is selectively taken up by the ZnuABC transporter that is active during zinc depletion, enhancing virulence.^166^ To overcome competition, other transporters, such as FoxA from *P. aeruginosa*, can also transport siderophores synthesized by other organisms (e.g., ferrioxamine and nocardiamine produced by *Streptomyces*) through a process called xenosiderophore utilization or siderophore “piracy”.^167–169^ Existing metal uptake/transport systems can also be used to sequester noniron metals even though they might not be as efficient.^170^ For example, *P. aeruginosa* exhibited 23–35% slower uptake rates for pyochelin–metal complexes with copper­(II), gallium­(III), manganese­(II), and nickel­(II) compared to that of ferric iron.^151,170^ Given the slow rates and reduced metal toxicity observed, pyochelin was suggested to play a role in heavy metal resistance, preventing additional toxic metals from diffusing across porins and accumulating within the cell.^151^ The import of pyochelin bound to noniron metals was found to be TonB-dependent, suggesting overlap with the iron acquisition pathway; however, the full mechanism of transport is not yet understood.^171^

Siderophore complexes have broader roles in metal homeostasis beyond iron acquisition from the environment.^172^ For example, auxin biosynthesis was promoted in several siderophore-producing strains of *Streptomyces* (*S. tendae* F4, *S. acidizcabies* E13, and *S. coelicolor* A3) grown in the presence of ferric iron as well as either aluminum­(II), cadmium­(II), copper­(II), or nickel­(II).^173^ Thus, these complexes can prevent metals from inhibiting the production of plant growth-promoting molecules, thereby increasing plant growth.^173^ Organisms that require specific metals can also use siderophores to select metal ions for metabolism.^174^ Some diazotrophs, *Azobacter vinelandii*, obtain key metals (e.g., molybdenum, vanadium, and iron) by using siderophores to sequester cofactors for functional nitrogenase enzymes.^174^ *A. vinelandii* was also shown to use siderophores to prevent the uptake of toxic metals, such as tungsten.^175^ There are several functions of siderophore noniron metal complexes within the environment and many more to be revealed.

### Challenges and New Strategies to Studying Bacteria from Metal-Rich Environments

Siderophore production in heavy metal-contaminated environments remains understudied due to limited access to these sites, the lack of robust cultivation methods for microbes from these extreme locales, and the development and affordability of molecular tools.^176,177^ Prior to the 2010s, tens of metagenomics papers were published annually on metal-polluted environments.^178^ Now hundreds of papers are published each year due to more affordable high-throughput sequencing with higher read density and increased read length per chip, as well as the use of nonstandard laboratory conditions for microbial cultivation.^179^ However, physical access to conduct research in some extreme locales remains a barrier.^180^ For example, in addition to the travel that may be required to access these locations, certain sites require permission, permits, and escorts to oversee fieldwork.^181^ Natural (e.g., mountains) and anthropogenic (e.g., hazardous waste sites) geological constraints are also key factors that limit access.^181^ These barriers restrict researchers from sampling these environments, leaving many open questions concerning how these organisms function and produce siderophores.^176^

Another significant challenge is that the vast majority of microbes are unculturable.^182^ Only 28% of bacterial species have 16S rRNA sequencing information available within public databases.^182^ Furthermore, microcolonies of culturable bacteria can have poor success rates of subcultivation.^182,183^ Whole full-length genomes are required to annotate metagenomic data, and finding complete genomes of heavy metal-resistant bacteria requires isolates to grow in stable and pure cultures.^184^ Reproducing native conditions, including temperature and pH, is not trivial, and other microbes in the environment can provide required substances, such as siderophores and other carbon sources, for the target organism to grow.^106,185^ Diffusion chambers have successfully cultivated previously unculturable bacteria from heavy metal-contaminated environments, such as nickel-contaminated soils.^186^ However, the unculturable pose major challenges to understanding bacterial metabolism, especially if bacteria express unknown physiological characteristics under various growth conditions.^187^

Without properly annotated genomes of metal-resistant microbes, we are unable to accurately analyze genomic sequence data, which is essential for taxonomic identification and functional characterization.^176^ While annotation tools are improving for genes unique to metal-rich environments, many sequences lack matches in databases due to divergent or novel lineages.^188–190^ Such divergence is what makes extremophile sequencing information so intriguing, as these organisms have evolved to have enzymes, metabolic processes, and additional natural products to give them competitive advantages.^191^ Moving forward, it is critical to develop sequence-based tools and culture methods that allow us to collect complete genomic data sets to identify key differences in these organisms.^176^

Several molecular techniques have provided insight into how to identify and cultivate the unculturable.^192,193^ Fluorescence in situ hybridization (FISH) is a molecular technique used to directly visualize microbial nucleotide sequences within the environment, which can be coupled with other techniques to aid in microbial isolation and cultivation.^194^ For example, in addition to using FISH, microarray filter devices and cell sorting techniques have been used to separate/dilute large quantities of marine bacteria to encourage cell growth up to 35%.^195^ Although FISH has been used to identify bacteria from metal-rich environments, such as acid mine drainage, there are no reports using this method to cultivate or find optimal growth conditions from these sites.^139,196,197^ However, FISH and cell sorting methods are still emerging in the field to detect, identify, and quantify microbes, but require more general tailoring for broader applications.^198^

Genomic sequencing provides information about metabolic pathways that can guide bacterial cultivation, such as insights into host, nutrient, and oxygen requirements, as well as antibiotic resistance.^184^ For instance, a nitrogen-fixing *Leptospirillum* was isolated from an acid mine drainage biofilm and determined to possess a *nifA* gene involved in the expression of nitrogen-fixing genes, which allowed for its successful cultivation in the absence of bioavailable nitrogen.^199^ This information, combined with predictive models of growth variables, can inform cultivation strategies.^200^ For example, a linear regression of genome-derived features has been used to predict the optimal growth temperatures for hundreds of bacteria within 5.18 °C.^200^ Other predictive models based on biogeographical data and taxon distribution, as well as amino acid composition and cellular localization, have also been developed to infer bacterial pH, oxygen, temperature, and salinity preferences.^201,202^

Single-cell genomics and transcriptomics can provide insight into cultivation conditions.^203^ These methods involve separating individual cells within environmental samples using microfluidic devices, amplifying their DNA or cDNA, and creating genomic libraries for sequencing.^203^ Although the use of single-cell techniques to cultivate microbes from metal-rich sites has yet to be reported, these methods can now yield medium to high-quality genomes or transcriptomes, improving the accuracy of sequence information.^204,205^ For example, Cross et al. used sequencing data to perform “reverse genomics”, where genes encoding membrane proteins unique to Saccharibacteria were identified and used to guide the generation of fluorescently labeled antibodies for cell sorting.^206^ Using human saliva samples, fluorescently labeled Saccharibacteria cells were sorted onto various agar plates, and colonies containing both Saccharibacteria and Actinobacteria were coisolated.^206^ This method required enriched syntrophic interactions, resulting in the cultivation of five new strains of Saccharibacteria.^206,207^

The quality of sequence data is another important factor for determining key requirements for bacterial growth in the laboratory.^208,209^ Sequence quality can be affected by sample preparation (e.g., nucleotide extraction), library construction, and sequencing methods.^210–215^ In metal-rich environments, there can be low biomass and metals that interfere with extraction methods, as well as downstream polymerase chain reactions; thus, additional extraction and cleaning steps may be required.^210,216^ Library construction may require more long, high-quality (c)­DNA or additional amplification steps that can be affected by metal contaminants inhibiting enzymatic steps or damaging (c)­DNA.^217^ Furthermore, GC-rich taxa can complicate constructing libraries, biasing sequence coverage.^218^ The choice of sequencing technology (e.g., Illumina short-read versus Pacific Biosciences or Oxford Nanopore Technology long-read methods) can also affect data quality, as high GC content and secondary structure can lead to low quality data and missing tandem repeats.^218,219^ Deep long-read sequencing and hybrid sequencing combining Illumina and either Pacific Biosciences or Oxford Nanopore Technology platforms can close gaps in short-read assemblies to obtain high-quality genomes.^139,220^ Long-read methods yield up to 10–12 kb average read lengths as opposed to the 100–300 bp reads from short-read sequencing methods, such as Illumina.^221^ Even though long-read methods are more expensive, have higher error rates, and require more time for data analysis, more publications are now using long-read sequencing to resolve long and complex repetitive elements.^222^

Top-down approaches using phenotype selection (e.g., metal resistance) followed by genomic sequencing can identify new taxa and functional genes, facilitating the annotation of genomes from these understudied environments.^208,223^ For example, gene-targeted sequencing, the amplification of target gene families using primers containing conserved sequences, can identify functional genes related to microbial phenotypes.^224^ “Focused identification of next-generation sequencing-based definitive enzyme research” or FINDER has been used to identify core metabolic genes and industrial enzymes in large metagenomic data sets from Indonesian hot springs, salt mines, and bogs using highly complementary sequences based on model organisms.^225^ The only caveat to methods such as FINDER is that sequence information resembling the metagenomes must be available to generate primers.^225^

Bottom-up approaches, such as function-based screening, have also been used to characterize DNA libraries, providing insight into gene function and annotation within these environments.^226^ For example, dual screens utilizing CAS and metal toxicity assays have been used to prioritize metal-resistant, metallophore-producing bacteria from Carpenter Snow Creek Site, a heavy metal-polluted site in Montana, USA.^154^ The genomes of these isolates were later sequenced, and extracts were analyzed by mass spectrometry, providing information linking genes to molecular phenotypes.^154^ While this strategy provides sequence information for genomes from heavy metal-contaminated environments, one drawback of bottom-up approaches is only finding homologues and not novel genes.^227^ However, to circumvent this limitation, techniques have been developed to identify novel genes with known functions in microbes. For example, eukaryotic RNA in metal-polluted Swedish sediment was used to make cDNA libraries that were screened for metal complementarity in metal-sensitive *S. cerevisiae*, revealing new classes of genes involved in metal homeostasis.^228^ Bottom-up approaches still leaves gaps in our understanding of entire genomes of nonmodel organisms; thus, integrating bottom-up with top-down approaches will likely provide more information on how to relate phenotypes to genotypes in metal-rich communities.^229^

### Applications of Siderophores

Having a better understanding of siderophore production in metal-rich environments will allow us to effectively leverage these metal chelators and their producers for novel applications in agriculture, bioremediation, medicine, and biometallurgy.^100^ Their relevance is especially important now, given increasing climate pressures on our food systems, and rising global populations increasing the demand for highly nutritional food and sustainable agricultural practices.^89^ Nutrient-deficient soil and other plant stressors (e.g., salinity, drought, altered nutrient cycling, or excess metals) can reduce crop yields.^230^ As such, siderophore-producing bacteria have been used to improve crop production.^231–233^ For example, bioinoculants of siderophore-producing *Pseudomonas*, *Aeromonas*, and *Aneurinibacillus aneurinilyticus* have been shown to increase the germination, root and shoot lengths, and overall weight of wheat.^231^ Plant growth can be enhanced using siderophore-iron complexes that increase iron bioavailability and phytohormones, such as indole-3-acetic acid.^232,233^ These molecules and their microbial producers are also a natural and less damaging alternative to chemical fertilizers in the remediation of contaminated soils.^234^ Siderophore-producing bacteria can be used as biocontrol agents by scavenging iron from plant pathogens, inhibiting their growth, and preventing disease.^232,235^ Pyoverdine secreted by several *Pseudomonas* can function as a natural pesticide against phytopathogens, such as *Xanthomonas oryzae* pv. oryzae, the causative agent of bacterial leaf blight.^185,236^ Furthermore, metal-resistant siderophore-producing rhizobacteria can also reduce the bioavailability of heavy metals in soils, resulting in the production of safer crops, as plants absorb less of these pollutants.^237^

Bioremediation practices can involve siderophores that chelate toxic metals, either immobilizing them or increasing their solubility to minimize metal toxicity and aid environmental cleanup.^238^ For example, yersiniabactin can adequately sequester both copper and zinc from water samples from Lake Erie, USA, as a potential water treatment solution.^239^ Although siderophore-producing fungi have been underreported in metal-rich environments, ferrichrome from *Aspergillus niger* has been used to extract or bioleach valuable uranium, thorium, and rare earth elements from phosphorites collected from Abu Tartur Mine in Egypt.^240^ Most bioremediation studies involving siderophores typically use siderophore-producing bacteria for metal extraction and removal.^241^ The pyoverdine-producing *P*. *fluorescens* from groundwater can also extract iron, uranium, and nickel from uranium ore mine samples.^242^ Arsenic-tolerant Actinobacteria have also been isolated from heavy metal-contaminated water in Saxony, Germany, and found to produce arsenic-binding siderophores that reduce metal availability.^243^ These examples are sustainable and cost-effective bioremediation practices compared to abiotic remediation methods, such as chemical neutralization, which are more costly and labor-intensive (Figure 4C).^244^ However, the large-scale field implementation of microbial bioremediation remains challenging due to several factors, including site heterogeneity, slow treatment rates, and chemical complexities.^245^

Phytoremediation is a form of bioremediation that uses plants to absorb and remove pollutants from the environment.^246^ In heavy metal-contaminated sites, metal hyperaccumulating plants, such as *Arabidopsis halleri*, are commonly used to remove metals from the environment and store them in organelles (e.g., protoplasts).^247,248^ The addition of siderophores, such as desferrioxamine B, can improve the phytoextraction of metals.^140^ For example, desferrioxamine B, in the presence of citric acid, improved the phytoextraction of germanium, lanthanum, europium, neodymium, and gadolinium.^140^ Siderophore-producing bacteria play a major role in phytoremediation, as these organisms are found in the rhizosphere, use siderophores to extract metal from the environment, and form metal–siderophore complexes that are taken up by plant roots (Figure 4C).^249^ For example, siderophore-producing *Agrobacterium* can remove arsenic from soil and increase the metal tolerance of poplar plants, promoting plant growth.^250^ When cadmium- and lead-tolerant pyoverdine-producing *P. putida* KNP9 was grown near mung bean plants, the plant size increased as the levels of each element in the roots were reduced by 50% and 90%, respectively.^251^ While the combination of phytoremediation and siderophore-producing bacteria is a sustainable method for removing pollutants, it can be dangerous if livestock consume metal hyperaccumulating plants.^252^ An ecological risk assessment of a phytoremediation study performed on an arsenic-contaminated site with the metal hyperaccumulator *Pteris cretica* revealed increased levels of arsenic in small mammals when siderophores were used as plant-growth promoters.^253^ Plants can also take an unreasonable amount of time to remediate metal pollution.^254^ A study showed that although corn and castor bean grown in metal-contaminated soil in the presence of chelator, nitrilotriacetic acid, removed 60 mmol kg^–1^ of heavy metal contaminants, and the total metalloid removal was estimated to take 6000 years.^255^ Furthermore, not all metals, such as mercury, can be hyperaccumulated by plants, likely due to poor solubility.^256^ Thus, phytoremediation efforts must strike a delicate balance between the efficiency of metal removal and overcoming the ecological risks associated with heavy metal mobility in nature.^253,254^

Siderophores also play major roles in modifying assemblages of microbial communities encased in extracellular polymeric substances that attach to surfaces called biofilms.^257^ Such aggregation impacts plant growth and is responsible for more than 80% of bacterial infections.^258^ Given that iron is an essential nutrient for cellular growth, siderophore-producing bacteria have shown promise as antibiofilm agents.^259^ For example, the TonB-dependent siderophore receptor gene was deleted from *P*. *fluorescens,* biofilm formation was significantly reduced (>40%); adenosine monophosphate was also determined to inhibit the receptor, reducing biofilm biomass.^173^ Increased *E. coli* biofilm formation was reported in the presence of the siderophore-uptake transporter gene *iroN*, suggesting that siderophores play an important role in producing biofilms.^260^ This correlation is further supported by *Stenotrophomonas maltophilia* rhizobacteria that produced elevated levels of biofilm in iron-deficient conditions.^261^ Siderophore-enriched biofilms can also increase plant growth through iron sequestration.^262^ For example, *B. velezensis* SQR9 has been reported to boost vegetable and grain yields by forming iron-enriched biofilms over plant roots.^262^ Combined with siderophore-producing bacteria, biofilms can also synergistically remove metals from the environment as they act as an additional barrier, restricting metal mobility.^263^ However, it remains unclear whether siderophore production contributes more significantly to the removal of metals than just extracellular polymeric substances.^264^

Aside from agricultural and medical use, siderophores have other unconventional applications beyond iron transport, such as the sustainable recovery of metals from non-natural environments.^265^ Metal leaching with bacteria, known as biometallurgy, is an important low-energy strategy for recovering valuable metals, such as lithium, cobalt, and nickel, found in electronics.^266^ Using conditions optimized for siderophore production in *P. aeruginosa,* researchers extracted 99% of lithium in just 6 days from cellphone lithium-ion batteries.^265^ Siderophores are being used to recover rare earth elements, which are in limited supply on the planet yet widely used in products that generate electronic waste,^267^ the fastest growing waste rate in the world to date.^268^ Desferrioxamine E has been used to extract indium from light-emitting device (LED) screens at a 32% yield in 90 days.^269^ While siderophores and siderophore-producing bacteria, especially metal-resistant bacteria from heavy metal contaminated environments, show promise in metal removal, they are not yet commercially used due to scalability, siderophore stability, and metal waste complexity.^249,270^ Further optimization and a deeper mechanistic understanding of siderophore–metal interactions are required to maximize the industrial potential of siderophores.^13^

## Conclusions

The role of siderophores in metal-rich environments is an active and expanding area of research that is currently not well understood beyond the context of iron-deficient conditions.^135^ While culture-independent methods circumvent cell cultivation to access taxonomic diversity and genes, culture-dependent methods are important to obtain more genetic information about how to annotate genes and understand gene function and metabolism in extreme environments.^184^ Both methods are necessary to gain insight into the diversity, genetic blueprints, and biochemistry of heavy metal-contaminated microbiomes.^192,271^ We look forward to reading future work that integrates these methods and uses other combinational approaches to discover new siderophores, elucidate their structures, and understand their roles within metal-rich ecosystems. This information will enable scientists to better exploit these molecules and metal-richlocales to improve human health and develop sustainable practices.

## References

[1] Kramer J., Özkaya Ö., Kümmerli R. (2020). Bacterial Siderophores in Community
and Host Interactions. Nat. Rev. Microbiol..

[2] Chen L., Li Z.-Y., Wang G.-Y. (2025). Siderophores Produced by Marine Microorganisms. Antonie Van Leeuwenhoek.

[3] Malik Z. I., Ghafoor M. U., Shah S. H. B. U., Abid J., Farooq U., Ahmad A. M. R. (2025). Unlocking Iron:
Nutritional Origins, Metabolic Pathways,
and Systemic Significance. Front. Nutr..

[4] Strzelecki P., Nowicki D. (2025). Tools to Study Microbial
Iron Homeostasis and Oxidative
Stress: Current Techniques and Methodological Gaps. Front. Mol. Biosci..

[5] Andrews S. C., Robinson A. K., Rodríguez-Quiñones F. (2003). Bacterial
Iron Homeostasis. FEMS Microbiol. Rev..

[6] Johnstone T. C., Nolan E. M. (2015). Beyond Iron: Non-Classical Biological
Functions of
Bacterial Siderophores. Dalton Trans..

[7] Sargun A., Gerner R. R., Raffatellu M., Nolan E. M. (2021). Harnessing Iron
Acquisition Machinery to Target *Enterobacteriaceae*. J. Infect. Dis..

[8] Harris W. R., Carrano C. J., Cooper S. R., Sofen S. R., Avdeef A. E., McArdle J. V., Raymond K. N. (1979). Coordination Chemistry of Microbial
Iron Transport Compounds. 19. Stability Constants and Electrochemical
Behavior of Ferric Enterobactin and Model Complexes. J. Am. Chem. Soc..

[9] Wilson B. R., Bogdan A. R., Miyazawa M., Hashimoto K., Tsuji Y. (2016). Siderophores in Iron Metabolism: From Mechanism to Therapy Potential. Trends Mol. Med..

[10] Choi S., Kronstad J. W., Jung W. H. (2024). Siderophore
Biosynthesis and Transport
Systems in Model and Pathogenic Fungi. J. Microbiol.
Biotechnol..

[11] Mayegowda S. B., Gadilingappa M. N. (2025). Microbial Siderophores: A New Insight on Healthcare
Applications. BME Front.

[12] Ghosh S. K., Bera T., Chakrabarty A. M. (2020). Microbial Siderophore – A
Boon to Agricultural Sciences. Biol. Control.

[13] Gräff Á.
T., Barry S. M. (2024). Siderophores
as Tools and Treatments. Npj Antimicrob. Resist..

[14] Mobarra N., Shanaki M., Ehteram H., Nasiri H., Sahmani M., Saeidi M., Goudarzi M., Pourkarim H., Azad M. (2016). A Review on Iron Chelators in Treatment
of Iron Overload Syndromes. Int. J. Hematol.-Oncol.
Stem Cell Res..

[15] Fan D., Fang Q. (2021). Siderophores for Medical Applications: Imaging, Sensors, and Therapeutics. Int. J. Pharm..

[16] Khasheii B., Mahmoodi P., Mohammadzadeh A. (2021). Siderophores: Importance in Bacterial
Pathogenesis and Applications in Medicine and Industry. Microbiol. Res..

[17] Koglin A., Walsh C. T. (2009). Structural Insights into Nonribosomal Peptide Enzymatic
Assembly Lines. Nat. Prod. Rep..

[18] Donfrancesco A., Deb G., Dominici C., Pileggi D., Castello M. A., Helson L. (1990). Effects of
a Single Course of Deferoxamine in Neuroblastoma Patients. Cancer Res..

[19] Golonka R., Yeoh B. S., Vijay-Kumar M. (2019). The Iron Tug-of-War between Bacterial
Siderophores and Innate Immunity. J. Innate
Immun..

[20] Wang J., Qian X.-Q., Yang T., Hou D.-B., Zhang G.-L., Li G.-Y. (2023). Chaetomadramines A–E, a Class of Siderophores with Potent
Neuroprotective Activity from the Fungus *Chaetomium madrasense* Cib-1. Fitoterapia.

[21] Virpiranta H., Banasik M., Taskila S., Leiviska T., Halttu M., Sotaniemi V.-H., Tanskanen J. (2020). Isolation
of Efficient Metal-Binding
Bacteria from Boreal Peat Soils and Development of Microbial Biosorbents
for Improved Nickel Scavenging. Water.

[22] Page M. G. P. (2019). The
Role of Iron and Siderophores in Infection, and the Development of
Siderophore Antibiotics. Clin. Infect. Dis..

[23] Pecoraro L., Wang X., Shah D., Song X., Kumar V., Shakoor A., Tripathi K., Ramteke P. W., Rani R. (2022). Biosynthesis
Pathways, Transport Mechanisms and Biotechnological Applications of
Fungal Siderophores. J. Fungi.

[24] Hesse E., O’Brien S., Tromas N., Bayer F., Luján A. M., van Veen E. M., Hodgson D. J., Buckling A. (2018). Ecological Selection
of Siderophore-Producing Microbial Taxa in Response to Heavy Metal
Contamination. Ecol. Lett..

[25] Yi X., Liang J.-L., Su J.-Q., Jia P., Lu J., Zheng J., Wang Z., Feng S., Luo Z., Ai H., Liao B., Shu W., Li J., Zhu Y.-G. (2022). Globally
Distributed Mining-Impacted Environments Are Underexplored Hotspots
of Multidrug Resistance Genes. ISME J..

[26] Ondrasek G., Shepherd J., Rathod S., Dharavath R., Rashid M. I., Brtnicky M., Shahid M. S., Horvatinec J., Rengel Z. (2025). Metal Contamination – a Global Environmental
Issue: Sources, Implications & Advances in Mitigation. RSC Adv..

[27] Roskova Z., Skarohlid R., McGachy L. (2022). Siderophores: An Alternative Bioremediation
Strategy?. Sci. Total Environ..

[28] Patel K. D., Fisk M. B., Gulick A. M. (2024). Discovery,
Functional Characterization,
and Structural Studies of the NRPS-Independent Siderophore Synthetases. Crit. Rev. Biochem. Mol. Biol..

[29] LeBlanc A. R., Wuest W. M. (2024). Siderophores: A
Case Study in Translational Chemical
Biology. Biochemistry.

[30] Springer S. D., Butler A. (2016). Microbial Ligand Coordination:
Consideration of Biological
Significance. Coord. Chem. Rev..

[31] Dell’Anno F., Vitale G. A., Buonocore C., Vitale L., Palma Esposito F., Coppola D., Della Sala G., Tedesco P., de Pascale D. (2022). Novel Insights
on Pyoverdine: From Biosynthesis to Biotechnological Application. Int. J. Mol. Sci..

[32] Wang M., Li H. (2025). Structure, Function, and Biosynthesis of Siderophores Produced by *Streptomyces* Species. J. Agric. Food
Chem..

[33] Hur G. H., Vickery C. R., Burkart M. D. (2012). Explorations of
Catalytic Domains
in Non-Ribosomal Peptide Synthetase Enzymology. Nat. Prod. Rep..

[34] Robbel L., Knappe T. A., Linne U., Xie X., Marahiel M. A. (2010). Erythrochelin
– a Hydroxamate-Type Siderophore Predicted from the Genome
of *Saccharopolyspora erythraea*. FEBS J..

[35] Hai Y., Jenner M., Tang Y. (2020). Fungal Siderophore
Biosynthesis Catalysed
by an Iterative Nonribosomal Peptide Synthetase. Chem. Sci..

[36] Jenner M., Hai Y., Nguyen H. H., Passmore M., Skyrud W., Kim J., Garg N. K., Zhang W., Ogorzalek Loo R. R., Tang Y. (2023). Elucidating the Molecular Programming of a Nonlinear Non-Ribosomal
Peptide Synthetase Responsible for Fungal Siderophore Biosynthesis. Nat. Commun..

[37] Condurso H. L., Bruner S. D. (2012). Structure and Noncanonical Chemistry
of Nonribosomal
Peptide Biosynthetic Machinery. Nat. Prod. Rep..

[38] Walsh C. T., Gehring A. M., Weinreb P. H., Quadri L. E., Flugel R. S. (1997). Post-Translational
Modification of Polyketide and Nonribosomal Peptide Synthases. Curr. Opin. Chem. Biol..

[39] Mordhorst S., Ruijne F., Vagstad A. L., Kuipers O. P., Piel J. (2023). Emulating
Nonribosomal Peptides with Ribosomal Biosynthetic Strategies. RSC Chem. Biol..

[40] Süssmuth R. D., Mainz A. (2017). Nonribosomal Peptide
Synthesis—Principles and Prospects. Angew.
Chem., Int. Ed..

[41] Miller D. A., Luo L., Hillson N., Keating T. A., Walsh C. T. (2002). Yersiniabactin Synthetase:
A Four-Protein Assembly Line Producing the Nonribosomal Peptide/Polyketide
Hybrid Siderophore of *Yersinia pestis*. Chem. Biol..

[42] Ronnebaum T. A., Lamb A. L. (2018). Nonribosomal Peptides for Iron Acquisition:
Pyochelin
Biosynthesis as a Case Study. Curr. Opin. Struct.
Biol..

[43] Raymond K. N., Dertz E. A., Kim S. S. (2003). Enterobactin: An Archetype for Microbial
Iron Transport. Proc. Natl. Acad. Sci. U. S.
A..

[44] Ahmadi M. K., Fawaz S., Jones C. H., Zhang G., Pfeifer B. A. (2015). Total Biosynthesis
and Diverse Applications of the Nonribosomal Peptide-Polyketide Siderophore
Yersiniabactin. Appl. Environ. Microbiol..

[45] Kadi N., Challis G. L. (2009). Chapter 17 Siderophore Biosynthesis: A Substrate Specificity
Assay for Nonribosomal Peptide Synthetase-Independent Siderophore
Synthetases Involving Trapping of Acyl-Adenylate Intermediates with
Hydroxylamine. Methods Enzymol..

[46] Giddings L.-A., Lountos G. T., Kim K. W., Brockley M., Needle D., Cherry S., Tropea J. E., Waugh D. S. (2021). Characterization
of a Broadly Specific Cadaverine *N*-Hydroxylase Involved
in Desferrioxamine B Biosynthesis in *Streptomyces sviceus*. PLoS One.

[47] Barona-Gómez F., Wong U., Giannakopulos A. E., Derrick P. J., Challis G. L. (2004). Identification
of a Cluster of Genes That Directs Desferrioxamine Biosynthesis in *Streptomyces coelicolor* M145. J. Am.
Chem. Soc..

[48] Oves-Costales D., Kadi N., Challis G. L. (2009). The Long-Overlooked Enzymology of
a Nonribosomal Peptide Synthetase-Independent Pathway for Virulence-Conferring
Siderophore Biosynthesis. Chem. Commun..

[49] Fisk M. B., Barrera Ramirez J., Merrick C. E., Wencewicz T. A., Gulick A. M. (2025). Identification and
Characterization of the Biosynthesis
of the Hybrid NRPS-NIS Siderophore Nocardichelin. ACS Chem. Biol..

[50] Manck L. E., Park J., Tully B. J., Poire A. M., Bundy R. M., Dupont C. L., Barbeau K. A. (2022). Petrobactin,
a Siderophore Produced
by *Alteromonas*, Mediates Community Iron Acquisition
in the Global Ocean. ISME J..

[51] Schnoes A. M., Brown S. D., Dodevski I., Babbitt P. C. (2009). Annotation Error
in Public Databases: Misannotation of Molecular Function in Enzyme
Superfamilies. PLOS Comput. Biol..

[52] Gu S., Shao Y., Rehm K., Bigler L., Zhang D., He R., Xu R., Shao J., Jousset A., Friman V.-P., Bian X., Wei Z., Kümmerli R., Li Z. (2024). Feature Sequence-Based
Genome Mining Uncovers the Hidden Diversity
of Bacterial Siderophore Pathways. eLife.

[53] Su L., Souaibou Y., Hôtel L., Paris C., Weissman K. J., Aigle B. (2024). Biosynthesis of Novel
Desferrioxamine Derivatives Requires Unprecedented
Crosstalk between Separate NRPS-Independent Siderophore Pathways. Appl. Environ. Microbiol..

[54] Blin K., Shaw S., Augustijn H. E., Reitz Z. L., Biermann F., Alanjary M., Fetter A., Terlouw B. R., Metcalf W. W., Helfrich E. J. N., van
Wezel G. P., Medema M. H., Weber T. (2023). antiSMASH
7.0: New and Improved Predictions for Detection, Regulation, Chemical
Structures and Visualisation. Nucleic Acids
Res..

[55] He R., Zhang J., Shao Y., Gu S., Song C., Qian L., Yin W.-B., Li Z. (2023). Knowledge-Guided Data
Mining on the Standardized Architecture of NRPS: Subtypes, Novel Motifs,
and Sequence Entanglements. PLOS Comput. Biol..

[56] Kang S.-M., Kang H.-S., Chung W.-H., Kang K.-T., Kim D.-H. (2024). Structural
Perspectives on Metal Dependent Roles of Ferric Uptake Regulator (Fur). Biomolecules.

[57] Miethke M., Marahiel M. A. (2007). Siderophore-Based Iron Acquisition and Pathogen Control. Microbiol. Mol. Biol. Rev..

[58] Escolar L., Pérez-Martín J., de Lorenzo V. (1999). Opening the
Iron Box: Transcriptional Metalloregulation by the Fur Protein. J. Bacteriol..

[59] Hantke K. (2001). Iron and Metal
Regulation in Bacteria. Curr. Opin. Microbiol..

[60] Merchant A.T., Spatafora G.A. (2014). A Role for the DtxR Family of Metalloregulators in
Gram-Positive Pathogenesis. Mol. Oral Microbiol..

[61] de
Peredo A. G., Saint-Pierre C., Latour J.-M., Michaud-Soret I., Forest E. (2001). Conformational Changes of the Ferric Uptake Regulation
Protein upon Metal Activation and DNA Binding; First Evidence of Structural
Homologies with the Diphtheria Toxin Repressor. J. Mol. Biol..

[62] Xiao J., Bu Y., Tao Y., Zhou Z., Zou J., Cui W., Yang Z. (2025). The Role of Bacterial Siderophores in Infection Therapy: From Anti-Infective
Mechanisms to Therapeutic Advances. Int. J.
Nanomedicine.

[63] de
Peredo A. G., Saint-Pierre C., Latour J.-M., Michaud-Soret I., Forest E. (2001). Conformational Changes of the Ferric Uptake Regulation
Protein upon Metal Activation and DNA Binding; First Evidence of Structural
Homologies with the Diphtheria Toxin Repressor. J. Mol. Biol..

[64] Okkotsu Y., Little A. S., Schurr M. J. (2014). The *Pseudomonas aeruginosa* AlgZR Two-Component System Coordinates Multiple Phenotypes. Front. Cell. Infect. Microbiol..

[65] Whistler C. A., Corbell N. A., Sarniguet A., Ream W., Loper J. E. (1998). The Two-Component
Regulators GacS and GacA Influence Accumulation of the Stationary-Phase
Sigma Factor ςS and the Stress Response in *Pseudomonas
fluorescens* Pf-5. J. Bacteriol..

[66] Dean C. R., Neshat S., Poole K. (1996). PfeR, an Enterobactin-Responsive
Activator of Ferric Enterobactin Receptor Gene Expression in *Pseudomonas aeruginosa*. J. Bacteriol..

[67] Salvado B., Vilaprinyo E., Sorribas A., Alves R. (2015). A Survey of HK, HPt,
and RR Domains and Their Organization in Two-Component Systems and
Phosphorelay Proteins of Organisms with Fully Sequenced Genomes. PeerJ..

[68] Mahren S., Braun V. (2003). The FecI Extracytoplasmic-Function
Sigma Factor of *Escherichia
coli* Interacts with the Β′ Subunit of RNA Polymerase. J. Bacteriol..

[69] Venturi V., Weisbeek P., Koster M. (1995). Gene Regulation
of Siderophore-Mediated
Iron Acquisition in *Pseudomonas*: Not Only the Fur
Repressor. Mol. Microbiol..

[70] Stiefel A., Mahren S., Ochs M., Schindler P. T., Enz S., Braun V. (2001). Control of the Ferric
Citrate Transport System of *Escherichia coli*: Mutations
in Region 2.1 of the FecI Extracytoplasmic-Function
Sigma Factor Suppress Mutations in the FecR Transmembrane Regulatory
Protein. J. Bacteriol..

[71] Saldaña-Ahuactzi Z., Knodler L. A. (2022). FoxR Is
an AraC-like Transcriptional Regulator of Ferrioxamine
Uptake in *Salmonella enterica*. Mol. Microbiol..

[72] Chareyre, S. ; Mandin, P. Bacterial Iron Homeostasis Regulation by sRNAs. Microbiol. Spectr. 2018, 6 (2). 10.1128/microbiolspec.RWR-0010-2017 PMC11633579 29573257

[73] Cornelis P., Tahrioui A., Lesouhaitier O., Bouffartigues E., Feuilloley M., Baysse C., Chevalier S. (2023). High Affinity
Iron Uptake by Pyoverdine in *Pseudomonas aeruginosa* Involves Multiple Regulators besides Fur, PvdS, and FpvI. BioMetals.

[74] Jaworska K., Konarska J., Gomza P., Rożen P., Nieckarz M., Krawczyk-Balska A., Brzostek K., Raczkowska A. (2023). Interplay
between the RNA Chaperone Hfq, Small RNAs and Transcriptional Regulator
OmpR Modulates Iron Homeostasis in the Enteropathogen *Yersinia
enterocolitica*. Int. J. Mol. Sci..

[75] Schalk I. J., Mislin G. L. A., Brillet K. (2012). Structure,
Function and Binding Selectivity
and Stereoselectivity of Siderophore-Iron Outer Membrane Transporters. Curr. Top. Membr..

[76] Xie B., Wei X., Wan C., Zhao W., Song R., Xin S., Song K. (2024). Exploring
the Biological Pathways of Siderophores and Their Multidisciplinary
Applications: A Comprehensive Review. Molecules.

[77] Clarke T. E., Ku S.-Y., Dougan D. R., Vogel H. J., Tari L. W. (2000). The Structure
of the Ferric Siderophore Binding Protein FhuD Complexed with Gallichrome. Nat. Struct. Biol..

[78] Mekuli R., Shoukat M., Dugat-Bony E., Bonnarme P., Landaud S., Swennen D., Hervé V. (2025). Iron-Based
Microbial Interactions:
The Role of Iron Metabolism in the Cheese Ecosystem. J. Bacteriol..

[79] Wang H., Fewer D. P., Holm L., Rouhiainen L., Sivonen K. (2014). Atlas of Nonribosomal Peptide and
Polyketide Biosynthetic
Pathways Reveals Common Occurrence of Nonmodular Enzymes. Proc. Natl. Acad. Sci. U. S. A..

[80] Klenotic P. A., Moseng M. A., Morgan C. E., Yu E. W. (2021). Structural and Functional
Diversity of RND Transporters. Chem. Rev..

[81] Faraldo-Gómez J. D., Sansom M. S. P. (2003). Acquisition of Siderophores in Gram-Negative Bacteria. Nat. Rev. Mol. Cell Biol..

[82] Liu L., Wang W., Wu S., Gao H. (2022). Recent Advances in
the Siderophore Biology of Shewanella. Front.
Microbiol..

[83] Imperi F., Tiburzi F., Visca P. (2009). Molecular
Basis of Pyoverdine Siderophore
Recycling in *Pseudomonas aeruginosa*. Proc. Natl. Acad. Sci. U. S. A..

[84] Stein N. V., Eder M., Burr F., Stoss S., Holzner L., Kunz H.-H., Jung H. (2023). The RND Efflux
System ParXY Affects
Siderophore Secretion in *Pseudomonas putida* KT2440. Microbiol. Spectr..

[85] Wells R. M., Jones C. M., Xi Z., Speer A., Danilchanka O., Doornbos K. S., Sun P., Wu F., Tian C., Niederweis M. (2013). Discovery of a Siderophore Export
System Essential
for Virulence of *Mycobacterium tuberculosis*. PLoS Pathog..

[86] Yeterian E., Martin L. W., Lamont I. L., Schalk I. J. (2010). An Efflux Pump Is
Required for Siderophore Recycling by *Pseudomonas aeruginosa*. Environ. Microbiol. Rep..

[87] Nishino K., Yamasaki S., Nakashima R., Zwama M., Hayashi-Nishino M. (2021). Function and
Inhibitory Mechanisms of Multidrug Efflux Pumps. Front. Microbiol..

[88] Postle K. (2007). TonB System, *In Vivo* Assays and Characterization. Methods Enzymol..

[89] Timofeeva A. M., Galyamova M. R., Sedykh S. E. (2022). Bacterial Siderophores: Classification,
Biosynthesis, Perspectives of Use in Agriculture. Plants.

[90] Celia H., Botos I., Ni X., Fox T., De Val N., Lloubes R., Jiang J., Buchanan S. K. (2019). Cryo-EM Structure
of the Bacterial Ton Motor Subcomplex ExbB–ExbD Provides Information
on Structure and Stoichiometry. Commun. Biol..

[91] Delepelaire P. (2019). Bacterial
ABC Transporters of Iron Containing Compounds. Res. Microbiol..

[92] Rees D. C., Johnson E., Lewinson O. (2009). ABC Transporters: The
Power to Change. Nat. Rev. Mol. Cell Biol..

[93] Ames G. F.-L. (1986). Bacterial
Periplasmic Transport Systems: Structure, Mechanism, and Evolution. Annu. Rev. Biochem..

[94] Chhabra R., Saha A., Chamani A., Schneider N., Shah R., Nanjundan M. (2020). Iron Pathways
and Iron Chelation
Approaches in Viral, Microbial, and Fungal Infections. Pharmaceuticals.

[95] Auda I. G., Ali Salman I. M., Odah J. Gh. (2020). Efflux Pumps of Gram-Negative Bacteria
in Brief. Gene Rep.

[96] Stintzi A., Barnes C., Xu J., Raymond K. N. (2000). Microbial
Iron Transport
via a Siderophore Shuttle: A Membrane Ion Transport Paradigm. Proc. Natl. Acad. Sci. U. S. A..

[97] Zhu W., Arceneaux J. E. L., Beggs M. L., Byers B. R., Eisenach K. D., Lundrigan M. D. (1998). Exochelin
Genes in *Mycobacterium smegmatis*: Identification
of an ABC Transporter and Two Non-Ribosomal Peptide
Synthetase Genes. Mol. Microbiol..

[98] Crouch M.-L. V., Castor M., Karlinsey J. E., Kalhorn T., Fang F. C. (2008). Biosynthesis
and IroC-Dependent Export of the Siderophore Salmochelin Are Essential
for Virulence of *Salmonella Enterica* serovar Typhimurium. Mol. Microbiol..

[99] Miethke M., Schmidt S., Marahiel M. A. (2008). The Major
Facilitator Superfamily-Type
Transporter YmfE and the Multidrug-Efflux Activator Mta Mediate Bacillibactin
Secretion in *Bacillus subtilis*. J. Bacteriol..

[100] Ahmed E., Holmström S. J.
M. (2014). Siderophores in Environmental
Research: Roles and Applications. Microb. Biotechnol..

[101] Fukushima T., Allred B. E., Sia A. K., Nichiporuk R., Andersen U. N., Raymond K. N. (2013). Gram-Positive Siderophore-Shuttle
with Iron-Exchange from Fe-Siderophore to Apo-Siderophore by *Bacillus cereus* YxeB. Proc. Natl.
Acad. Sci. U. S. A..

[102] Taylor, T. A. ; Unakal, C. G. *Staphylococcus aureus* Infection. In StatPearls; StatPearls Publishing: Treasure Island, FL, 2025. 28722898

[103] Himpsl S. D., Mobley H. L. T. (2019). Siderophore Detection Using Chrome
Azurol S and Cross-Feeding Assays. Methods Mol.
Biol. Clifton NJ..

[104] Reitz Z. L., Medema M. H. (2022). Genome Mining Strategies for Metallophore
Discovery. Curr. Opin. Biotechnol..

[105] Gomes A. F. R., Sousa E., Resende D. I. S. P. (2024). A
Practical
Toolkit for the Detection, Isolation, Quantification, and Characterization
of Siderophores and Metallophores in Microorganisms. ACS Omega.

[106] D’Onofrio A., Crawford J. M., Stewart E. J., Witt K., Gavrish E., Epstein S., Clardy J., Lewis K. (2010). Siderophores
from Neighboring Organisms Promote the Growth of Uncultured Bacteria. Chem. Biol..

[107] Louden B. C., Haarmann D., Lynne A. M. (2011). Use of
Blue Agar
CAS Assay for Siderophore Detection. J. Microbiol.
Biol. Educ. JMBE.

[108] Patel P. R., Shaikh S. S., Sayyed R. Z. (2018). Modified Chrome
Azurol S Method for Detection and Estimation of Siderophores Having
Affinity for Metal Ions Other than Iron. Environ.
Sustain.

[109] Pérez-Miranda S., Cabirol N., George-Téllez R., Zamudio-Rivera L. S., Fernández F. J. (2007). O-CAS, a Fast and Universal Method
for Siderophore Detection. J. Microbiol. Methods.

[110] Csáky T. Z., Hassel O., Rosenberg T., Lång S., Turunen E., Tuhkanen A. (1948). On the Estimation of
Bound Hydroxylamine in Biological Materials. Acta Chem. Scand..

[111] Arnow L. E. (1937). Colorimetric Determination Of The
Components Of 3,4-Dihydroxyphenylalaninetyrosine
Mixtures. J. Biol. Chem..

[112] Shenker M., Oliver I., Helmann M., Hadar Y., Chen Y. (1992). Utilization by Tomatoes of Iron Mediated
by a Siderophore Produced
by *Rhizopus arrhizus*. J. Plant
Nutr..

[113] Wang Y., Huang W., Li Y., Yu F., Penttinen P. (2022). Isolation, Characterization, and Evaluation of a High-Siderophore-Yielding
Bacterium from Heavy Metal–Contaminated Soil. Environ. Sci. Pollut. Res..

[114] Rai V., Fisher N., Duckworth O. W., Baars O. (2020). Extraction and Detection
of Structurally Diverse Siderophores in Soil. Front. Microbiol..

[115] Kundu K., Teta R., Esposito G., Stornaiuolo M., Costantino V. (2023). A Four-Step Platform to Optimize Growth Conditions
for High-Yield Production of Siderophores in Cyanobacteria. Metabolites.

[116] Kreutzer F., Nett M. (2012). Genomics-Driven Discovery of Taiwachelin,
a Lipopeptide Siderophore from *Cupriavidus taiwanensis*. Org. Biomol. Chem..

[117] He R., Gu S., Xu J., Li X., Chen H., Shao Z., Wang F., Shao J., Yin W.-B., Qian L., Wei Z., Li Z. (2024). SIDERITE:
Unveiling
Hidden Siderophore Diversity in the Chemical Space through Digital
Exploration. iMeta.

[118] Falcao B. P., Di Matteo V., Hrouzek P., Štenclová L., Urajová P., Mareš J., Kuta J., Yerena J. A. M., Kozlíková-Zapomělová E., Esposito G., Mangoni A., Costantino V., Galica T. (2025). Cyanochelin B: A Cyanobacterium-Produced Siderophore
with Photolytic Properties That Negate Iron Monopolization in UV Light. Appl. Environ. Microbiol..

[119] Schlüter L., Hansen K. Ø., Isaksson J., Andersen J. H., Hansen E. H., Kalinowski J., Schneider Y. K.-H. (2024). Discovery
of Thiazostatin D/E Using UPLC-HR-MS2-Based Metabolomics and σ-Factor
Engineering of *Actinoplanes* sp. SE50/110. Front. Bioeng. Biotechnol..

[120] Aron A. T., Petras D., Schmid R., Gauglitz J. M., Büttel I., Antelo L., Zhi H., Nuccio S.-P., Saak C. C., Malarney K. P., Thines E., Dutton R. J., Aluwihare L. I., Raffatellu M., Dorrestein P. C. (2022). Native
Mass Spectrometry-Based Metabolomics Identifies Metal-Binding Compounds. Nat. Chem..

[121] D’Argenio V. (2018). The High-Throughput Analyses Era:
Are We Ready for
the Data Struggle?. High-Throughput.

[122] Ten K. E., Rahman S., Tan H. S. (2025). Transcriptomic
Insights
into the Virulence of *Acinetobacter baumannii* during
Infection—Role of Iron Uptake and Siderophore Production Genes. FEBS Lett..

[123] Gerlt J. A., Bouvier J. T., Davidson D. B., Imker H. J., Sadkhin B., Slater D. R., Whalen K. L. (2015). Enzyme Function
Initiative-Enzyme Similarity Tool (EFI-EST): A Web Tool for Generating
Protein Sequence Similarity Networks. Biochim.
Biophys. Acta.

[124] Ellermann M., Arthur J. C. (2017). Siderophore-Mediated
Iron Acquisition
and Modulation of Host-Bacterial Interactions. Free Radic. Biol. Med..

[125] Feng Y., Kong L., Zheng R., Wu X., Zhou J., Xu X., Liu S. (2024). Adjusted Bacterial
Cooperation in Anammox Community to Adapt to High Ammonium in Wastewater
Treatment Plant. Water Res. X.

[126] Dong Z., Yu M., Cai Y., Ma Y., Chen Y., Hu B. (2023). Directed Regulation of Anammox Communities
Based on Exogenous Siderophores for Highly Efficient Nitrogen Removal. Water Res..

[127] Zheng R., Kong L., Feng Y., Chen B., Gu Y., Wu X., Liu S. (2025). Siderophore-Mediated Cooperation
in Anammox Consortia. Environ. Sci. Technol..

[128] Kramer J., Özkaya Ö., Kümmerli R. (2020). Bacterial
Siderophores in Community and Host Interactions. Nat. Rev. Microbiol..

[129] Gaddy J. A., Arivett B. A., McConnell M. J., López-Rojas R., Pachón J., Actis L. A. (2012). Role of Acinetobactin-Mediated
Iron Acquisition Functions in the Interaction of *Acinetobacter
baumannii* Strain ATCC 19606T with Human Lung Epithelial Cells,
Galleria Mellonella Caterpillars, and Mice. Infect. Immun..

[130] Rayner B., Verderosa A. D., Ferro V., Blaskovich M. A. T. (2023). Siderophore
Conjugates to Combat Antibiotic-Resistant Bacteria. RSC Med. Chem..

[131] De Serrano L. O., Camper A. K., Richards A. M. (2016). An Overview
of Siderophores
for Iron Acquisition in Microorganisms Living in the Extreme. BioMetals.

[132] Marschner, H. Functions of Mineral Nutrients: Macronutrients. In Marschner’s Mineral Nutrition of Higher Plants; Elsevier, 2002; pp 229–312. 10.1016/B978-0-08-057187-4.50014-X

[133] Singh P., Chauhan P. K., Upadhyay S. K., Singh R. K., Dwivedi P., Wang J., Jain D., Jiang M. (2022). Mechanistic
Insights and Potential Use of Siderophores Producing Microbes in Rhizosphere
for Mitigation of Stress in Plants Grown in Degraded Land. Front. Microbiol..

[134] Vijay K., Shibasini M., Sivasakthivelan P., Kavitha T. (2023). Microbial Siderophores
as Molecular Shuttles for Metal
Cations: Sources, Sinks and Application Perspectives. Arch. Microbiol..

[135] Gomes A. F. R., Almeida M. C., Sousa E., Resende D. I. S. P. (2024). Siderophores
and Metallophores: Metal Complexation Weapons to Fight Environmental
Pollution. Sci. Total Environ..

[136] Wołowiec M., Komorowska-Kaufman M.łg., Pruss A., Rzepa G., Bajda T. (2019). Removal of Heavy Metals
and Metalloids from Water Using Drinking
Water Treatment Residuals as Adsorbents: A Review. Minerals.

[137] Briffa J., Sinagra E., Blundell R. (2020). Heavy Metal Pollution
in the Environment and Their Toxicological Effects on Humans. Heliyon.

[138] Li B., Ming H., Qin S., Nice E. C., Dong J., Du Z., Huang C. (2025). Redox Regulation: Mechanisms,
Biology and Therapeutic
Targets in Diseases. Signal Transduct. Target.
Ther..

[139] Giddings L.-A., Kunstman K., Moumen B., Asiama L., Green S., Delafont V., Brockley M., Samba-Louaka A. (2022). Isolation
and Genome Analysis of an Amoeba-Associated Bacterium *Dyella
terrae* Strain Ely Copper Mine From Acid Rock Drainage in
Vermont, United States. Front. Microbiol..

[140] Bruins M. R., Kapil S., Oehme F. W. (2000). Microbial
Resistance
to Metals in the Environment. Ecotoxicol. Environ.
Saf..

[141] Schweitzer, P. N. Active Mines and Mineral Processing Plants in the United States in 2003; U.S. Geological Survey, 2020. https://mrdata.usgs.gov/metadata/mineplant.faq.html#what.1 (accessed 2026-02-04).

[142] Schweitzer, P. N. Rare earth element mines, deposits, and occurrences; U.S. Geological Survey, 2020. https://mrdata.usgs.gov/metadata/of02-189.faq.html#what.1 (accessed 2026-02-04).

[143] Thisani S. K., Kallon D. V. V., Byrne P. (2020). Geochemical Classification
of Global Mine Water Drainage. Sustainability.

[144] Baker B. J., Banfield J. F. (2003). Microbial Communities
in Acid Mine
Drainage. FEMS Microbiol. Ecol..

[145] Huang Y., Li X.-T., Jiang Z., Liang Z.-L., Wang P., Liu Z.-H., Li L.-Z., Yin H.-Q., Jia Y., Huang Z.-S., Liu S.-J., Jiang C.-Y. (2021). Key Factors Governing
Microbial Community in Extremely Acidic Mine Drainage (pH < 3). Front. Microbiol..

[146] Skeba S., Snyder M., Maltman C. (2023). Metallophore Activity
toward the Rare Earth Elements by Bacteria Isolated from Acid Mine
Drainage Due to Coal Mining. Microorganisms.

[147] Mghazli N., Sbabou L., Hakkou R., Ouhammou A., El Adnani M., Bruneel O. (2021). Description of Microbial
Communities
of Phosphate Mine Wastes in Morocco, a Semi-Arid Climate, Using High-Throughput
Sequencing and Functional Prediction. Front.
Microbiol..

[148] Schalk I. J., Hannauer M., Braud A. (2011). New Roles
for Bacterial
Siderophores in Metal Transport and Tolerance. Environ. Microbiol..

[149] Bau M., Tepe N., Mohwinkel D. (2013). Siderophore-Promoted
Transfer of
Rare Earth Elements and Iron from Volcanic Ash into Glacial Meltwater,
River and Ocean Water. Earth Planet. Sci. Lett..

[150] Kraemer D., Kopf S., Bau M. (2015). Oxidative
Mobilization
of Cerium and Uranium and Enhanced Release of “Immobile”
High Field Strength Elements from Igneous Rocks in the Presence of
the Biogenic Siderophore Desferrioxamine B. Geochim. Cosmochim. Acta.

[151] Braud A., Geoffroy V., Hoegy F., Mislin G. L. A., Schalk I. J. (2010). Presence of the Siderophores Pyoverdine and Pyochelin
in the Extracellular Medium Reduces Toxic Metal Accumulation in *Pseudomonas aeruginosa* and Increases Bacterial Metal Tolerance. Environ. Microbiol. Rep..

[152] Tircsó G., Garda Z., Kálmán F. K., Baranyai Z., Pócsi I., Balla G., Tóth I. (2013). Lanthanide­(III)
Complexes of Some Natural Siderophores: A Thermodynamic, Kinetic and
Relaxometric Study. J. Inorg. Biochem..

[153] Kraemer D., Bau M. (2022). Siderophores and the
Formation of
Cerium Anomalies in Anoxic Environments. Geochem.
Perspect. Lett..

[154] Ahmed M. M. A., Hammers C., Boudreau P. D. (2024). Dual Screen for
Metal-Tolerant Metallophore Producers Evaluated with Soil from the
Carpenter Snow Creek Site, a Heavy-Metal-Toxified Site in Montana. ACS Omega.

[155] Morey J. R., Kehl-Fie T. E. (2020). Bioinformatic Mapping
of Opine-Like
Zincophore Biosynthesis in Bacteria. mSystems.

[156] Johnston C. W., Wyatt M. A., Li X., Ibrahim A., Shuster J., Southam G., Magarvey N. A. (2013). Gold Biomineralization
by a Metallophore from a Gold-Associated Microbe. Nat. Chem. Biol..

[157] Maldonado-Hernández J., Román-Ponce B., Arroyo-Herrera I., Guevara-Luna J., Ramos-Garza J., Embarcadero-Jiménez S., Estrada de los Santos P., Wang E. T., Vásquez-Murrieta M. S. (2022). Metallophores Production
by Bacteria Isolated from Heavy Metal-Contaminated Soil and Sediment
at Lerma–Chapala Basin. Arch. Microbiol..

[158] Shil H., Sharma P., Gola R. (2025). Exploring
Copper-Resistant
Bacterial Diversity in Coal Mines: Implications for Environmental
Bioremediation. Geomicrobiol. J..

[159] Crits-Christoph A., Bhattacharya N., Olm M. R., Song Y. S., Banfield J. F. (2021). Transporter Genes
in Biosynthetic Gene Clusters Predict
Metabolite Characteristics and Siderophore Activity. Genome Res..

[160] Giddings L.-A., Chlipala G., Kunstman K., Green S., Morillo K., Bhave K., Peterson H., Driscoll H., Maienschein-Cline M. (2020). Characterization of an Acid Rock Drainage Microbiome
and Transcriptome at the Ely Copper Mine Superfund Site. PLoS One.

[161] Kondakindi V. R., Pabbati R., Erukulla P., Maddela N. R., Prasad R. (2024). Bioremediation of Heavy Metals-Contaminated
Sites by
Microbial Extracellular Polymeric Substances – A Critical View. Environ. Chem. Ecotoxicol..

[162] Schwabe R., Senges C. H. R., Bandow J. E., Heine T., Lehmann H., Wiche O., Schlömann M., Levicán G., Tischler D. (2020). Cultivation Dependent Formation of
Siderophores by *Gordonia Rubripertincta* CWB2. Microbiol. Res..

[163] Ochoa-Agudelo S., Bedoya-Vélez J.
M., Villa-Restrepo A. F., Osorio-Tobón J. F. (2025). Lead Tolerance and Siderophore Production
by Native *Pseudomonas* spp. Isolated from Lead-Contaminated
Environments in Colombia. Folia Microbiol. (Praha).

[164] Gaonkar T., Bhosle S. (2013). Effect of Metals on a Siderophore
Producing Bacterial Isolate and Its Implications on Microbial Assisted
Bioremediation of Metal Contaminated Soils. Chemosphere.

[165] Schwabe R., Senges C. H. R., Bandow J. E., Heine T., Lehmann H., Wiche O., Schlömann M., Levicán G., Tischler D. (2020). Data on Metal-Chelating, -Immobilisation
and Biosorption Properties by *Gordonia rubripertincta* CWB2 in Dependency on Rare Earth Adaptation. Data Brief.

[166] Desrosiers D. C., Bearden S. W., Mier I., Abney J., Paulley J. T., Fetherston J. D., Salazar J. C., Radolf J. D., Perry R. D. (2010). Znu Is the Predominant
Zinc Importer in *Yersinia
pestis* during *In Vitro* Growth but Is Not
Essential for Virulence. Infect. Immun..

[167] Will V., Frey C., Normant V., Kuhn L., Chicher J., Volck F., Schalk I. J. (2024). The Role of FoxA,
FiuA, and FpvB in Iron Acquisition via Hydroxamate-Type Siderophores
in *Pseudomonas aeruginosa*. Sci. Rep..

[168] Josts I., Veith K., Tidow H. (2019). Ternary Structure of
the Outer Membrane Transporter FoxA with Resolved Signalling Domain
Provides Insights into TonB-Mediated Siderophore Uptake. eLife.

[169] Kumar R., Singh A., Srivastava A. (2025). Xenosiderophores:
Bridging the Gap in Microbial Iron Acquisition Strategies. World J. Microbiol. Biotechnol..

[170] Braud A., Hannauer M., Mislin G. L. A., Schalk I. J. (2009). The *Pseudomonas aeruginosa* Pyochelin-Iron
Uptake Pathway and
Its Metal Specificity. J. Bacteriol..

[171] Ducret V., Gonzalez D., Perron K. (2023). Zinc Homeostasis in *Pseudomonas*. BioMetals.

[172] Arnold E. (2024). Non-Classical Roles of Bacterial
Siderophores in Pathogenesis. Front. Cell. Infect.
Microbiol..

[173] Dimkpa C. O., Svatoš A., Dabrowska P., Schmidt A., Boland W., Kothe E. (2008). Involvement
of Siderophores
in the Reduction of Metal-Induced Inhibition of Auxin Synthesis in *Streptomyces* spp. Chemosphere.

[174] McRose D. L., Baars O., Morel F. M. M., Kraepiel A. M. L. (2017). Siderophore
Production in *Azotobacter vinelandii* in Response
to Fe-, Mo- and V-Limitation. Environ. Microbiol..

[175] Wichard T., Bellenger J.-P., Loison A., Kraepiel A. M. L. (2008). Catechol
Siderophores Control Tungsten Uptake and Toxicity in the Nitrogen-Fixing
Bacterium. Azotobacter vinelandii. Environ.
Sci. Technol..

[176] Rampelotto P. H. (2024). Extremophiles
and Extreme Environments: A Decade of
Progress and Challenges. Life.

[177] Tighe S., Afshinnekoo E., Rock T. M., McGrath K., Alexander N., McIntyre A., Ahsanuddin S., Bezdan D., Green S. J., Joye S., Stewart
Johnson S., Baldwin D. A., Bivens N., Ajami N., Carmical J. R., Herriott I. C., Colwell R., Donia M., Foox J., Greenfield N., Hunter T., Hoffman J., Hyman J., Jorgensen E., Krawczyk D., Lee J., Levy S., Garcia-Reyero N., Settles M., Thomas K., Gómez F., Schriml L., Kyrpides N., Zaikova E., Penterman J., Mason C. E. (2017). Genomic Methods and Microbiological
Technologies for Profiling Novel and Extreme Environments for the
Extreme Microbiome Project (XMP). J. Biomol.
Technol. JBT.

[178] Offiong N.-A. O., Edet J. B., Shaibu S. E., Akan N. E., Atakpa E. O., Sanganyado E., Okop I. J., Benson N. U., Okoh A. (2023). Metagenomics: An Emerging Tool for the Chemistry of Environmental
Remediation. Front. Environ. Chem..

[179] Teufel M., Sobetzko P. (2022). Reducing Costs for
DNA and RNA Sequencing
by Sample Pooling Using a Metagenomic Approach. BMC Genomics.

[180] Rampelotto P. H. (2024). Extremophiles
and Extreme Environments: A Decade of
Progress and Challenges. Life.

[181] Whiting S. N., Reeves R. D., Richards D., Johnson M. S., Cooke J. A., Malaisse F., Paton A., Smith J. a. C., Angle J. S., Chaney R. L., Ginocchio R., Jaffré T., Johns R., McIntyre T., Purvis O. W., Salt D. E., Schat H., Zhao F. J., Baker A. J. M. (2004). Research
Priorities for Conservation of Metallophyte Biodiversity and Their
Potential for Restoration and Site Remediation. Restor. Ecol..

[182] Hofer U. (2018). The Majority Is Uncultured. Nat. Rev. Microbiol..

[183] Zheng M., Wen L., He C., Chen X., Si L., Li H., Liang Y., Zheng W., Guo F. (2024). Sequencing-Guided
Re-Estimation and Promotion of Cultivability for Environmental Bacteria. Nat. Commun..

[184] Liu S., Moon C. D., Zheng N., Huws S., Zhao S., Wang J. (2022). Opportunities and Challenges
of Using Metagenomic Data to Bring Uncultured
Microbes into Cultivation. Microbiome.

[185] Saha M., Sarkar S., Sarkar B., Sharma B. K., Bhattacharjee S., Tribedi P. (2016). Microbial Siderophores
and Their
Potential Applications: A Review. Environ. Sci.
Pollut. Res..

[186] Remenár M., Karelová E., Harichová J., Zámocký M., Kamlárová A., Ferianc P. (2015). Isolation of Previously Uncultivable Bacteria from
a Nickel Contaminated Soil Using a Diffusion-Chamber-Based Approach. Appl. Soil Ecol..

[187] Gutleben J., Chaib De Mares M., van Elsas J. D., Smidt H., Overmann J., Sipkema D. (2018). The Multi-Omics Promise
in Context: From Sequence to Microbial Isolate. Crit. Rev. Microbiol..

[188] Chen J., Gao X., Zhang C., Ge Y. (2025). Rapid Identification
of Metal Resistance Genes Using an Enhanced ResNet Deep Learning Model
Trained on a Largely Expanded BacMet-Based Database. J. Hazard. Mater..

[189] Pal C., Bengtsson-Palme J., Rensing C., Kristiansson E., Larsson D. G. J. (2014). BacMet: Antibacterial Biocide and Metal Resistance
Genes Database. Nucleic Acids Res..

[190] Rappaport H. B., Oliverio A. M. (2023). Extreme Environments Offer an Unprecedented
Opportunity to Understand Microbial Eukaryotic Ecology, Evolution,
and Genome Biology. Nat. Commun..

[191] Salwan R., Sharma V. (2022). Genomics of Prokaryotic
Extremophiles
to Unfold the Mystery of Survival in Extreme Environments. Microbiol. Res..

[192] Rafiq M., Hassan N., Rehman M., Hayat M., Nadeem G., Hassan F., Iqbal N., Ali H., Zada S., Kang Y., Sajjad W., Jamal M. (2023). Challenges
and Approaches of Culturing the Unculturable Archaea. Biology.

[193] Stewart E. J. (2012). Growing Unculturable Bacteria. J. Bacteriol..

[194] Moter A., Göbel U. B. (2000). Fluorescence *in Situ* Hybridization (FISH) for Direct Visualization of
Microorganisms. J. Microbiol. Methods.

[195] Connon S. A., Giovannoni S. J. (2002). High-Throughput Methods for Culturing
Microorganisms in Very-Low-Nutrient Media Yield Diverse New Marine
Isolates. Appl. Environ. Microbiol..

[196] Bond P. L., Druschel G. K., Banfield J. F. (2000). Comparison of Acid
Mine Drainage Microbial Communities in Physically and Geochemically
Distinct Ecosystems. Appl. Environ. Microbiol..

[197] Edwards K. J., Gihring T. M., Banfield J. F. (1999). Seasonal Variations
in Microbial Populations and Environmental Conditions in an Extreme
Acid Mine Drainage Environment. Appl. Environ.
Microbiol..

[198] Kuhn T. M., Paulsen M., Cuylen-Haering S. (2024). Accessible
High-Speed Image-Activated Cell Sorting. Trends
Cell Biol..

[199] Tyson G. W., Lo I., Baker B. J., Allen E. E., Hugenholtz P., Banfield J. F. (2005). Genome-Directed Isolation of the
Key Nitrogen Fixer *Leptospirillum ferrodiazotrophum* sp. nov. from an Acidophilic Microbial Community. Appl. Environ. Microbiol..

[200] Sauer D. B., Wang D.-N. (2019). Predicting the Optimal
Growth Temperatures
of Prokaryotes Using Only Genome Derived Features. Bioinformatics.

[201] Ramoneda J., Stallard-Olivera E., Hoffert M., Winfrey C. C., Stadler M., Niño-García J. P., Fierer N. (2023). Building a
Genome-Based Understanding of Bacterial pH Preferences. Sci. Adv..

[202] Barnum T. P., Crits-Christoph A., Molla M., Carini P., Lee H. H., Ostrov N. (2024). Predicting
Microbial Growth Conditions
from Amino Acid Composition. bioRxiv.

[203] Kalisky T., Blainey P., Quake S. R. (2011). Genomic Analysis
at the Single-Cell Level. Annu. Rev. Genet..

[204] Liu S., Trapnell C. (2016). Single-Cell Transcriptome
Sequencing: Recent Advances
and Remaining Challenges. F1000Research.

[205] Gupta P., O’Neill H., Wolvetang E. J., Chatterjee A., Gupta I. (2024). Advances in Single-Cell
Long-Read
Sequencing Technologies. NAR Genomics Bioinforma.

[206] Cross K. L., Campbell J. H., Balachandran M., Campbell A. G., Cooper C. J., Griffen A., Heaton M., Joshi S., Klingeman D., Leys E., Yang Z., Parks J. M., Podar M. (2019). Targeted Isolation and Cultivation
of Uncultivated Bacteria by Reverse Genomics. Nat. Biotechnol..

[207] Lewis W. H., Ettema T. J. G. (2019). Culturing the
Uncultured. Nat. Biotechnol..

[208] Garza D. R., Dutilh B. E. (2015). From Cultured to
Uncultured Genome
Sequences: Metagenomics and Modeling Microbial Ecosystems. Cell. Mol. Life Sci. CMLS.

[209] Ha C. W. Y., Devkota S. (2020). The New Microbiology:
Cultivating
the Future of Microbiome-Directed Medicine. Am. J. Physiol. - Gastrointest. Liver Physiol..

[210] Foysal M. J., Salgar-Chaparro S. J. (2024). Improving
the Efficiency of DNA Extraction
from Iron Incrustations and Oilfield-Produced Water. Sci. Rep..

[211] Child H. T., Wierzbicki L., Joslin G. R., Tennant R. K. (2024). Comparative
Evaluation of Soil DNA Extraction Kits for Long Read Metagenomic Sequencing. Access Microbiol.

[212] Barber D. G., Davies C. A., Hartley I. P., Tennant R. K. (2024). Evaluation
of Commercial RNA Extraction Kits for Long-Read Metatranscriptomics
in Soil. Microb. Genomics.

[213] Head S. R., Komori H. K., LaMere S. A., Whisenant T., Van Nieuwerburgh F., Salomon D. R., Ordoukhanian P. (2014). Library Construction
for Next-Generation Sequencing: Overviews and Challenges. BioTechniques.

[214] Gaulke C. A., Schmeltzer E. R., Dasenko M., Tyler B. M., Vega Thurber R., Sharpton T. J. (2021). Evaluation of the Effects of Library
Preparation Procedure and Sample Characteristics on the Accuracy of
Metagenomic Profiles. mSystems.

[215] Cheng C., Fei Z., Xiao P. (2023). Methods to
Improve
the Accuracy of Next-Generation Sequencing. Front. Bioeng. Biotechnol..

[216] Herrera A., Cockell C. S. (2007). Exploring Microbial Diversity in
Volcanic Environments: A Review of Methods in DNA Extraction. J. Microbiol. Methods.

[217] Bonsu D. N. O., Higgins D., Simon C., Henry J. M., Austin J. J. (2024). Metal–DNA Interactions: Exploring
the Impact
of Metal Ions on Key Stages of Forensic DNA Analysis. Electrophoresis.

[218] Browne P. D., Nielsen T. K., Kot W., Aggerholm A., Gilbert M. T. P., Puetz L., Rasmussen M., Zervas A., Hansen L. H. (2020). GC Bias Affects Genomic and Metagenomic
Reconstructions, Underrepresenting GC-Poor Organisms. GigaScience.

[219] Spealman P., Burrell J., Gresham D. (2020). Inverted Duplicate
DNA Sequences Increase Translocation Rates through Sequencing Nanopores
Resulting in Reduced Base Calling Accuracy. Nucleic Acids Res..

[220] Chi B.-B., Lu Y.-N., Yin P.-C., Liu H.-Y., Chen H.-Y., Shan Y. (2021). Sequencing and Comparative
Genomic
Analysis of a Highly Metal-Tolerant *Penicillium janthinellum* P1 Provide Insights Into Its Metal Tolerance. Front. Microbiol..

[221] Goodwin S., McPherson J. D., McCombie W. R. (2016). Coming of Age: Ten
Years of next-Generation Sequencing Technologies. Nat. Rev. Genet..

[222] Iyer S. V., Goodwin S., McCombie W. R. (2024). Leveraging the Power
of Long Reads for Targeted Sequencing. Genome
Res..

[223] Herrera-Calderon A. C., Leal L., Suárez-Bautista J. D., Manotas-Viloria H. S., Muñoz-García A., Franco D., Arenas N. E., Vanegas J. (2024). Metagenomic and Genomic Analysis
of Heavy Metal-Tolerant and -Resistant Bacteria in Resource Islands
in a Semi-Arid Zone of the Colombian Caribbean. Environ. Sci. Pollut. Res. Int..

[224] Suenaga H. (2015). Targeted Metagenomics Unveils the
Molecular Basis for
Adaptive Evolution of Enzymes to Their Environment. Front. Microbiol..

[225] Sung J.-Y., Lee Y.-J., Cho Y.-J., Shin M.-N., Lee S.-J., Lee H.-S., Koh H., Bae J.-W., Shin J.-H., Kim H. J., Lee D.-W. (2021). A Large-Scale Metagenomic
Study for Enzyme Profiles Using the Focused Identification of the
NGS-Based Definitive Enzyme Research (FINDER) Strategy. Biotechnol. Bioeng..

[226] Delavat F., Phalip V., Forster A., Plewniak F., Lett M.-C., Lièvremont D. (2012). Amylases without
Known Homologues
Discovered in an Acid Mine Drainage: Significance and Impact. Sci. Rep..

[227] Weisman C. M., Murray A. W., Eddy S. R. (2020). Many, but Not All,
Lineage-Specific Genes Can Be Explained by Homology Detection Failure. PLoS Biol..

[228] Lehembre F., Doillon D., David E., Perrotto S., Baude J., Foulon J., Harfouche L., Vallon L., Poulain J., Da Silva C., Wincker P., Oger-Desfeux C., Richaud P., Colpaert J. V., Chalot M., Fraissinet-Tachet L., Blaudez D., Marmeisse R. (2013). Soil Metatranscriptomics
for Mining Eukaryotic Heavy Metal Resistance Genes. Environ. Microbiol..

[229] Shahzad K., J. Loor J. (2012). Application of Top-Down
and Bottom-up
Systems Approaches in Ruminant Physiology and Metabolism. Curr. Genomics.

[230] Ma Y., Dias M. C., Freitas H. (2020). Drought and
Salinity Stress Responses
and Microbe-Induced Tolerance in Plants. Front.
Plant Sci..

[231] Kumar P., Thakur S., Dhingra G. K., Singh A., Pal M. K., Harshvardhan K., Dubey R. C., Maheshwari D. K. (2018). Inoculation
of Siderophore Producing Rhizobacteria and Their Consortium for Growth
Enhancement of Wheat Plant. Biocatal. Agric.
Biotechnol..

[232] Zhao L., Wang Y., Kong S. (2020). Effects of *Trichoderma asperellum* and Its Siderophores on Endogenous
Auxin in *Arabidopsis thaliana* under Iron-Deficiency
Stress. Int. Microbiol..

[233] Jinal H. N., Gopi K., Prittesh P., Kartik V. P., Amaresan N. (2019). Phytoextraction of Iron from Contaminated
Soils by
Inoculation of Iron-Tolerant Plant Growth-Promoting Bacteria in Brassica
Juncea L. Czern. Environ. Sci. Pollut. Res..

[234] de Andrade L. A., Santos C. H. B., Frezarin E. T., Sales L. R., Rigobelo E. C. (2023). Plant Growth-Promoting
Rhizobacteria for Sustainable
Agricultural Production. Microorganisms.

[235] Deb C. R., Tatung M. (2024). Siderophore Producing
Bacteria as
Biocontrol Agent against Phytopathogens for a Better Environment:
A Review. South Afr. J. Bot..

[236] Liu Y., Dai C., Zhou Y., Qiao J., Tang B., Yu W., Zhang R., Liu Y., Lu S.-E. (2021). Pyoverdines Are
Essential for the Antibacterial Activity of *Pseudomonas chlororaphis* YL-1 under Low-Iron Conditions. Appl. Environ.
Microbiol..

[237] Singh, S. K. ; Singh, P. P. ; Gupta, A. ; Singh, A. K. ; Keshri, J. Tolerance of Heavy Metal Toxicity Using PGPR Strains of *Pseudomonas* Species. In PGPR Amelioration in Sustainable Agriculture; Singh, A. K. , Kumar, A. , Singh, P. K. , Eds.; Woodhead Publishing, 2019; pp 239–252. 10.1016/B978-0-12-815879-1.00012

[238] Newsome L., Falagán C. (2021). The Microbiology
of Metal Mine Waste:
Bioremediation Applications and Implications for Planetary Health. GeoHealth.

[239] Ahmadi M. K., Ghafari M., Atkinson J. D., Pfeifer B. A. (2016). A Copper
Removal Process for Water Based upon Biosynthesis of Yersiniabactin,
a Metal-Binding Natural Product. Chem. Eng.
J..

[240] Osman Y., Gebreil A., Mowafy A. M., Anan T. I., Hamed S. M. (2019). Characterization
of *Aspergillus niger* Siderophore That Mediates Bioleaching
of Rare Earth Elements from
Phosphorites. World J. Microbiol. Biotechnol..

[241] O’Brien S., Hodgson D. J., Buckling A. (2014). Social Evolution
of
Toxic Metal Bioremediation in *Pseudomonas aeruginosa*. Proc. R. Soc. B Biol. Sci..

[242] Edberg F., Kalinowski B. E., Holmström S. J. M., Holm K. (2010). Mobilization of Metals
from Uranium Mine Waste: The
Role of Pyoverdines Produced by *Pseudomonas fluorescens*. Geobiology.

[243] Retamal-Morales G., Mehnert M., Schwabe R., Tischler D., Zapata C., Chávez R., Schlömann M., Levicán G. (2018). Detection of Arsenic-Binding Siderophores
in Arsenic-Tolerating
Actinobacteria by a Modified CAS Assay. Ecotoxicol.
Environ. Saf..

[244] Atuchin V. V., Asyakina L. K., Serazetdinova Y. R., Frolova A. S., Velichkovich N. S., Prosekov A. Yu (2023). Microorganisms for
Bioremediation of Soils Contaminated with Heavy Metals. Microorganisms.

[245] Kuppan N., Padman M., Mahadeva M., Srinivasan S., Devarajan R. (2024). A Comprehensive Review of Sustainable
Bioremediation
Techniques: Eco Friendly Solutions for Waste and Pollution Management. Waste Manag. Bull..

[246] Sharma J. K., Kumar N., Singh N. P., Santal A. R. (2023). Phytoremediation
Technologies and Their Mechanism for Removal of Heavy Metal from Contaminated
Soil: An Approach for a Sustainable Environment. Front. Plant Sci..

[247] Kajala K., Walker K. L., Mitchell G. S., Krämer U., Cherry S. R., Brady S. M. (2019). Real-time Whole-plant Dynamics of
Heavy Metal Transport in *Arabidopsis halleri* and *Arabidopsis thaliana* by Gamma-ray Imaging. Plant Direct.

[248] Cosio C., Martinoia E., Keller C. (2004). Hyperaccumulation of
Cadmium and Zinc in *Thlaspi caerulescens* and *Arabidopsis halleri* at the Leaf Cellular Level. Plant Physiol.

[249] Rajkumar M., Ae N., Prasad M. N. V., Freitas H. (2010). Potential
of Siderophore-Producing Bacteria for Improving Heavy Metal Phytoextraction. Trends Biotechnol.

[250] Wang Q., Xiong D., Zhao P., Yu X., Tu B., Wang G. (2011). Effect of Applying an Arsenic-resistant
and Plant Growth–Promoting
Rhizobacterium to Enhance Soil Arsenic Phytoremediation by *Populus deltoides* LH05–17. J. Appl. Microbiol..

[251] Tripathi M., Munot H. P., Shouche Y., Meyer J. M., Goel R. (2005). Isolation and Functional Characterization
of Siderophore-Producing
Lead- and Cadmium-Resistant *Pseudomonas putida* KNP9. Curr. Microbiol..

[252] Kafle A., Timilsina A., Gautam A., Adhikari K., Bhattarai A., Aryal N. (2022). Phytoremediation: Mechanisms, Plant
Selection and Enhancement by Natural and Synthetic Agents. Environ. Adv..

[253] Jeong S., Moon H. S., Nam K. (2015). Increased Ecological
Risk Due to the Hyperaccumulation of As in *Pteris cretica* during the Phytoremediation of an As-Contaminated Site. Chemosphere.

[254] Yan A., Wang Y., Tan S. N., Mohd Yusof M. L., Ghosh S., Chen Z. (2020). Phytoremediation: A
Promising Approach
for Revegetation of Heavy Metal-Polluted Land. Front. Plant Sci..

[255] da Silva W. R., da Silva F. B. V., Araújo P. R. M., do Nascimento C. W. A. (2017). Assessing Human Health Risks and
Strategies for Phytoremediation in Soils Contaminated with As, Cd,
Pb, and Zn by Slag Disposal. Ecotoxicol. Environ.
Saf..

[256] Moreno F. N., Anderson C. W. N., Stewart R. B., Robinson B. H., Ghomshei M., Meech J. A. (2005). Induced Plant Uptake and Transport
of Mercury in the Presence of Sulphur-Containing Ligands and Humic
Acid. New Phytol..

[257] Singh R., Paul D., Jain R. K. (2006). Biofilms:
Implications
in Bioremediation. Trends Microbiol.

[258] Dhivya R., Rajakrishnapriya V. C., Sruthi K., Chidanand D. V., Sunil C. K., Rawson A. (2022). Biofilm Combating
in the Food Industry:
Overview, Non-Thermal Approaches, and Mechanisms. J. Food Process. Preserv..

[259] Shen T., Cao C., Zhu R., Chen J., Wang F., Wang Y. (2025). Identification of a
TonB-Dependent
Siderophore Receptor as a Novel Anti-Biofilm Target and Virtual Screening
for Its Inhibitor in *Pseudomonas fluorescens* PF08. Foods.

[260] Ballén V., Gabasa Y., Ratia C., Sánchez M., Soto S. (2022). Correlation Between Antimicrobial Resistance, Virulence Determinants
and Biofilm Formation Ability Among Extraintestinal Pathogenic *Escherichia coli* Strains Isolated in Catalonia, Spain. Front. Microbiol..

[261] Kalidasan V., Joseph N., Kumar S., Awang Hamat R., Neela V. K. (2018). Iron and Virulence in *Stenotrophomonas maltophilia*: All We Know So Far. Front. Cell. Infect.
Microbiol..

[262] Tan T., Xu Z., Tao L., Sun X., Xie J., Miao Y., Zhang N., Xun W., Beauregard P. B., Kovács Á.
T., Yu Y., Luo Y., Ran W., Zhang R., Shen Q. (2025). Siderophore-Mediated
Iron Enrichment
in the Biofilm Matrix Enhances Plant Iron Nutrition. Cell Rep.

[263] Ali A., Zahra A., Kamthan M., Husain F. M., Albalawi T., Zubair M., Alatawy R., Abid M., Noorani M. S. (2023). Microbial
Biofilms: Applications, Clinical Consequences, and Alternative Therapies. Microorganisms.

[264] Balíková K., Vojtková H., Duborská E., Kim H., Matúš P., Urík M. (2022). Role of Exopolysaccharides of *Pseudomonas* in Heavy Metal Removal and Other Remediation Strategies. Polymers.

[265] Vishwakarma A., Hait S. (2024). Selective Lithium Extraction from
Spent Lithium-Ion Batteries Using Siderophores Produced by *Pseudomonas aeruginosa*: Efficacy, Kinetics, and Artificial
Neural Network Modeling. Process Biochem..

[266] Zhang X., Shi H., Tan N., Zhu M., Tan W., Daramola D., Gu T. (2023). Advances in Bioleaching
of Waste
Lithium Batteries under Metal Ion Stress. Bioresour.
Bioprocess..

[267] Gulliani S., Volpe M., Messineo A., Volpe R. (2023). Recovery of
Metals and Valuable Chemicals from Waste Electric and Electronic Materials:
A Critical Review of Existing Technologies. RSC Sustain.

[268] Barshai, A. Global E-waste Statistics. emew, 2025.https://emew.com/blog/global-e-waste-statistics (accessed 2025-10-28).

[269] Zheng K., Benedetti M. F., Jain R., Guy B. M., Pollmann K., van Hullebusch E. D. (2024). Selective Leaching of Indium from
Spent LCD Screens by Siderophore Desferrioxamine E. J. Hazard. Mater..

[270] Bahaloo-Horeh N., Sadri F. (2025). Advancements in Siderophore-Based
Technologies for Metal Biorecovery. Hydrometallurgy.

[271] Yang Z., Lian Z., Liu L., Fang B., Li W., Jiao J. (2023). Cultivation Strategies
for Prokaryotes from Extreme
Environments. iMeta.

